# Population-Predicted MHC Class II Epitope Presentation of SARS-CoV-2 Structural Proteins Correlates to the Case Fatality Rates of COVID-19 in Different Countries

**DOI:** 10.3390/ijms22052630

**Published:** 2021-03-05

**Authors:** Chunguang Liang, Elena Bencurova, Eric Psota, Priya Neurgaonkar, Martina Prelog, Carsten Scheller, Thomas Dandekar

**Affiliations:** 1 Department of Bioinformatics, Biocenter, Am Hubland, University of Würzburg, 97074 Würzburg, Germany; liang@biozentrum.uni-wuerzburg.de (C.L.); elena.bencurova@uni-wuerzburg.de (E.B.); priya.neurgaonkar@uni-wuerzburg.de (P.N.); 2 Department of Pediatrics, Rheumatology and Special Immunology, University Hospital Würzburg, Pediatric Rheumatology/Special Immunology, Josef-Schneider-Str. 2, 97080 Würzburg, Germany; eric.psota@hotmail.de (E.P.); Prelog_M@ukw.de (M.P.); 3 Institute of Virology and Immunobiology, University of Würzburg, Versbacher Str. 7, 97078 Würzburg, Germany

**Keywords:** COVID-19, population coverage, MHC II, MHC I, B-cell, T-cell, epitope mapping, lethality rate, infection spread, SARS-CoV-2

## Abstract

We observed substantial differences in predicted Major Histocompatibility Complex II (MHCII) epitope presentation of SARS-CoV-2 proteins for different populations but only minor differences in predicted MHCI epitope presentation. A comparison of this predicted epitope MHC-coverage revealed for the early phase of infection spread (till day 15 after reaching 128 observed infection cases) highly significant negative correlations with the case fatality rate. Specifically, this was observed in different populations for MHC class II presentation of the viral spike protein (*p*-value: 0.0733 for linear regression), the envelope protein (*p*-value: 0.023), and the membrane protein (*p*-value: 0.00053), indicating that the high case fatality rates of COVID-19 observed in some countries seem to be related with poor MHC class II presentation and hence weak adaptive immune response against these viral envelope proteins. Our results highlight the general importance of the SARS-CoV-2 structural proteins in immunological control in early infection spread looking at a global census in various countries and taking case fatality rate into account. Other factors such as health system and control measures become more important after the early spread. Our study should encourage further studies on MHCII alleles as potential risk factors in COVID-19 including assessment of local populations and specific allele distributions.

## 1. Introduction

Since early 2020, SARS-CoV-2, which causes COVID-19, developed into a global pandemic. Many potential approaches to treatment and prophylaxis have been developed over a relatively short period. Currently, 238 vaccine candidates are in pre-clinical and clinical development (WHO, listing as of 2 February 2021) and six are at the same time already used worldwide (Oxford/AstraZeneca, Pfizer/BioNTech, Sinovac, Sinopharm, Moderna, and Sputnik V). However, most of the treatment agents show disputable efficacy or side effects [1]. Understanding immunological recognition and presentation of epitopes is a key step in controlling and combating viral diseases. Accordingly, several experimental and computerized models can be used to cover the areas of epidemiology, drug repurposing and vaccine design [2]. One of the most challenging approaches is to identify T and B cell epitopes that are correlating with optimal CD4+ helper, CD8+ cytotoxic T cell and B cell responses and may be associated with clinically mild COVID-19 courses or even constitute potential vaccine candidates [3,4,5]. However, one of the main problems is the presence of diverse circulating SARS-CoV-2 variants, and the diversity of Major Histocompatibility Complex (MHC) class I and class II alleles within the human population worldwide [6]. Presentation at MHCI and MHCII is critical for a sufficient activation of cytotoxic and helper T cell reactivity as well as for B cell stimulation and marks the potential of the adaptive immune system to establish a strong and long-lasting immune memory relevant to set up herd immunity and for the efficacy of future vaccine candidates.

The spread of viral diseases is influenced by several factors, such as the movements of the human population, social behavior, virus mutations and population immunity. Therefore, to understand the country-specific responses elicited by the MHCI and MHCII repertoires, population data on the spread of COVID-19 infection and the calculated lethality should be correlated with predictions of the immunological repertoire in a particular population.

To address this issue, our analysis considers: (i) infection spread and pattern of COVID-19-patient growth curves in different countries; (ii) focus on naïve population and natural immunity, defined for the time period from disease-free population till day 15 after reaching 128 infected cases in the population and (iii) country-specific human host immune response according to epitope response reaction frequencies separated for T- and B-cells (major eliciting MHCII epitopes mapped on SARS-CoV-2 surface epitopes).

In this paper, we establish and present several correlations between pandemic spread in different countries and the immune response to epitope-representation in immunogenic SARS-CoV-2 proteins. Full data of a broad analysis including different parameter choices are given (see also supplement), including negative controls. Potential limitations of this study and other factors such as control measures, health system, strain variation and local immunity are discussed. These immunological observations and correlations should now be further investigated together with probing of molecular features of the COVID-19 infection. Particularly in the early phase of the COVID-19 infection clear and strong correlations for MHC class I and II representation and case fatality rate become apparent while further factors change this in later time points.

## 2. Results

### 2.1. Prediction of B- and T-Cell Epitopes

Protein annotation and protein–protein interactions with the human host did provide some hints on SARS-CoV-2 infection biology and treatment strategies. However, there are some lacunas in the methods and in the analyses provided to date. Therefore, we decided to take the analysis one decisive step further to in silico predictions of potential linear B-cell epitopes. These analyses were performed with several online tools to rule out bias from individual prediction algorithms. The number of predicted epitopes significantly varied between the analyzed proteins.

For the analysis of B-cell epitopes, three distinct methods were used. The methods include ABCpre, BepiPred and IEDB. These methods work on discrete principles which help to explore various aspects of epitopes. Therefore the results are in varied combinations which can be further processed and interpreted. For example, the ABCpred method predicted a high number of epitopes with the default parameters. However, compared to BepiPred, IEDB analysis revealed unique epitopes which are neither predicted by BepiPred nor ABCpred. The BepiPred is one of the most popular tools for linear B-cell analysis. We compared the epitopes predicted in this study with experimentally verified epitopes, and most of them also matched (Figure S1). All the tools used in this analysis are set at their default parameters.

The non-structural protein ORF1ab was evaluated as the most antigenic protein. However, due to its nature and length, it is controversial to consider ORF1ab as a vaccine candidate. On the other hand, the ORF1ab encodes viral enzymes crucial for viral replication, such as viral protease (position: 1564–3882 AA, see Table S1) that contains several highly antigenic regions and is also a drug target. Spike protein (surface glycoprotein) is generally considered to be highly antigenic therefore represents a significant potential target for the vaccine [7,8]. Mapping of spike protein revealed 115 B-cell epitopes predicted by IEDB, 28 by BepiPred-2.0 and 117 was determined by ABCpred (Table 1). The full list of predicted B-cell epitopes is listed in Table S2.

To compare our findings with already experimentally validated epitopes, we selected epitope ^786^QILPDPLKPTKRSFIEDLLFNKVTLA^811^, which can induce the production of neutralizing antibodies in SARS-CoV-2 infected patients [9]. Interestingly, this epitope was predicted by ABCpred (as 10-mer starting at position 789) and BepiPred-2.0 (predicted as two epitopes, as 14-mer at 786 AA, and 9-mer ^806^LPDPSKPSKR^815^). The predictions by IEDB are overlapping 7-mers starting at 782 AA.

Several studies have recently been published on the bioinformatics prediction of B-cell epitope prediction in SARS-CoV-2 proteins. A summary of these articles is summarized in Noorimotlagh et al. 2020 [3]. In the work of Wang et al. (2020), a structure-based analysis was performed to select the most promising spike protein epitope for vaccine development [10]. Their analysis identified nine linear B-cell epitopes, which we compared with the results of our analysis. Two epitopes were identical with epitopes found by both BepiPred and ABCpred (epitopes at positions 441–448 and 657–664). One epitope starting at position 696 was found also by both tools, however, only in the truncated or extended form and five epitopes (CVNLTTRTQLPPAYTNS, VTWFHAIHVSGTNG, LGVYYHKNNKSW, TPINLVRDLPQGF and DEVRQIAPGQTGKI) were found exclusively using BepiPred. None of the epitopes was identical to the epitopes retrieved by IEDB analysis. On the other hand, compared to the work of Grifoni et al. (2020), our results were clearly different [11]. Of the 29 linear B-cell epitopes described by Grifoni, only two were identical to the epitopes predicted by BepiPred (11-mer at position 65, eight-mer at position 1157) and one epitope each by ABCpred (^280^NENGTITDA^288^) and IEDB (^1229^MVTIMLCCMTS^1239^).

Also, we found several similarities between already validated epitopes of previous SARS-CoV and novel SARS-CoV-2 epitopes, however, the amino acid sequences of these viruses are not identical, and thus several differences in predicted epitopes were observed (Figure S1). A similar observation was noted in Grifoni et al. (2020), which compared the amino acid sequence of SARS-CoV-2 and three related coronaviruses, Bat-SL-CoV, SARS-CoV and MERS-CoV. Their findings indicate that there are relatively high levels of similarity between SARS-CoV, Bat-SL-CoV and SARS-CoV-2, but only low sequence similarities regarding MERS-CoV [11]. Likewise, Ahmed et al. (2020) found high similarities of SARS-CoV-2 and SARS-CoV structural proteins, but MERS-CoV proteins shared less than 46% similarities with SARS-CoV-2 [7].

Identification of T-cell epitopes was performed using the analysis resource TepiTool (IEDB). We included the 27 most frequent alleles for the MHCI and the 26 most frequent alleles of MHC class II. Interestingly, multiple alleles of each MHCI and MHCII bind the same epitopes or overlap. For example, we found 16 epitopes in OFR10, of which only two (YINVFAFPF and MGYINVFAF) bind exclusively MHCI. The number of epitopes predicted in silico for each structural protein is given in Table 2. The full list of T-cell epitopes with associated alleles and IC50 values is available in the supplement (Table S3).

Each of the viral proteins was further analyzed by the VaxiJen server (threshold 0.4, Table 1). Proteins have potential antigenicity ranging from 0.4624 to 0.7185 indicating high antigenicity for each protein. The most antigenic protein is ORF10 (score 0.7158), however, protein does not have an exposed transmembrane domain and it is probably not folded into protein [12,13]. The role of ORF10 is not fully understood, but it probably interacts with cullin-2 RING E3 ligase complex, which mediates the degradation of restriction factors [14,15]. The highest VaxiJen score of structural proteins was noted for envelope protein (0.6025), followed by the membrane (0.5102) and nucleocapsid protein (0.5059) and finally the surface (spike) protein with a score of 0.4646. Each of the analyzed proteins has an antigenicity score above the threshold, so there is a high possibility that they can interact with MHC alleles to induce the immune response.

### 2.2. T-Cell Epitope Distribution

The frequency of expression of Human Leukocyte Antigen (HLA) alleles is crucial for understanding the spread of disease, and the pathogenicity of viruses, bacteria and parasites. MHC molecules are highly polymorphic and provide information on how the patients will respond to an antigen [16]. Allelic distribution significantly depends on the ethnic and geographical origin of the population [17]. Population coverage analysis can therefore help to find novel treatment targets regarding vaccine action specific for the susceptible group of inhabitants.

The population coverage analysis was performed by testing in silico predicted epitopes and their recognizing HLA alleles using the IEDB Population coverage analysis tool [16]. For analysis of MHCI distribution, we selected 76 epitopes for surface (spike) protein, 30 for membrane, 20 for the envelope and 18 for nucleocapsid protein. The forty-six epitopes of surface glycoprotein (spike), 12 epitopes of envelope and membrane proteins and eleven nucleocapsid epitopes were selected for the analysis of MHCII alleles distribution (Table S4).

**Figure 1:**
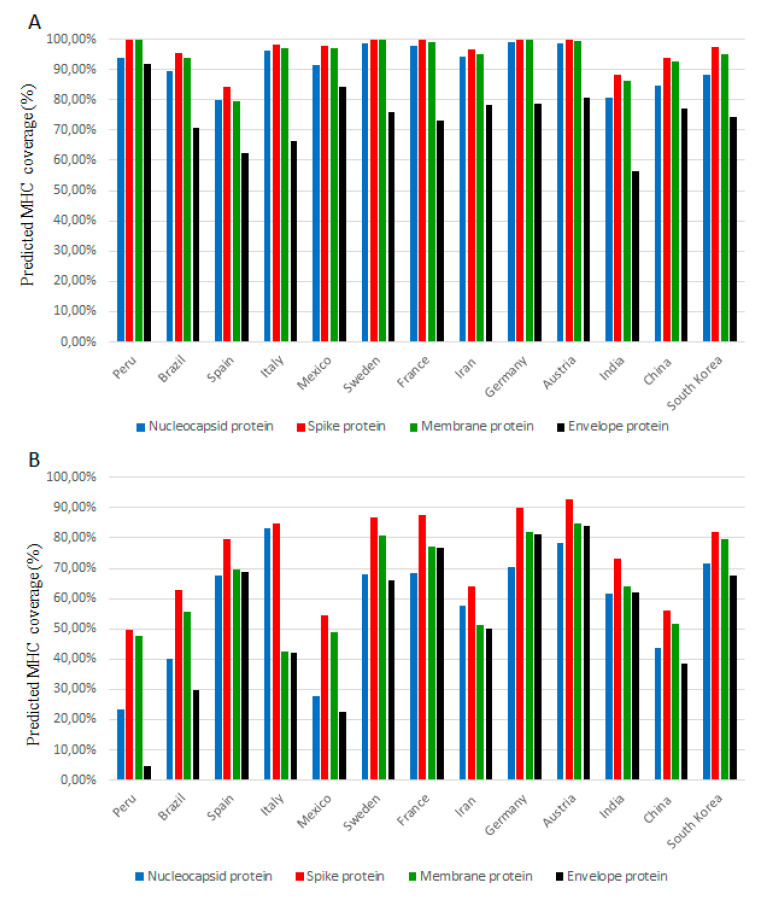
The predicted population coverage of most frequent Major Histocompatibility Complex (MHC) class I (**A**) and MHC class II (**B**) epitopes (predicted in silico) of SARS-CoV-2 structural proteins. The representation of MHC class I and II allele distribution in selected countries. The *y*-axis shows the percentage of population coverage according to the IEDB tool. The complete data are listed in Supplementary Table S5. Specific controls include results and tool validation on other viruses (Table S6) as well as results for scrambled sequences (Table S7).

We observed significant differences in the coverage of MHC alleles by SARS-CoV-2 proteins for different populations. Figure 1 shows the distribution of epitope-recognizing alleles in some of the most affected countries (India, France, Mexico, Peru, Brazil, Italy, Spain, Iran and China) and countries with lower reporting rate (Finland, South Korea, Sweden, Austria and Germany). The complete set of results for the whole world are given in suppl. Table S5. The highest population coverage for MHCI alleles was observed in European countries geographically located in the northwest; Finland—more than 99% for each nucleocapsid, membrane and spike proteins, 84.44% for envelope protein; England—more than 99% for each nucleocapsid, membrane and spike proteins, 80.02% for envelope protein; Ireland—more than 99% for each nucleocapsid, membrane and spike proteins, 79.94% for envelope protein, followed by Australia, Germany, Austria and Sweden (Figure 1 panel A, Table S5). The lowest distribution of MHCI allele was predicted for Latin American countries (Venezuela, Colombia, and Guatemala) and the United Arab Emirates and Wales, however, these results are likely to be inaccurate due to limited population data from the Allele Frequency database.

Similar results were observed for MHCII alleles (Figure 1, panel B, Table S5). The highest coverage for spike-recognizing alleles was detected in Ireland (94.03% coverage), Norway (93.75%) and England (92.88%), while Austria (84.95%), Norway (84.79%) and England (83.74%) were found as countries with highest alleles coverage for membrane proteins. A lower number of alleles recognized the epitopes on the surface protein. We found the highest coverage in Austria (84.16%), followed by Norway (84%), Ireland (83.10%), England (82.50%), and Germany (80.98%). Surprisingly, the highest coverage of in silico predicted epitopes for nucleocapsid proteins was observed in Italy (83.10%), Ethiopia (79.44%) and Austria (78.19%). Despite the high predicted coverage in Italy, the country was one of the most affected during the outbreak in the spring of 2020. In this context, it is necessary to note, that the binding affinity between the predicted epitope and recognizing allele does not fully reflect the T-cell response of individuals. As a negative control, the scrambled sequence of each structural protein was generated and the “population coverage” of the scrambled sequence was performed with the same parameters as for the viral protein (Table S7).

An important factor in understanding the prevalence of SARS-CoV-2 is the analysis of different ethnicities. MHC molecules are highly polymorphic and their frequencies vary in different ethnical groups [16]. These differences are the main reason for different susceptibility; however, several other factors have already been identified, including co-morbidities and access to medical care [18]. According to the available data, we selected different ethnic groups living in the United States to compare the T-cell response (Figure S2). The lowest binding abilities in both predetermined MHC classes were observed in the Indigenous people, Austronesians and Black Americans. It is noteworthy that these ethnic groups suffered from high mortality (published elsewhere [19,20,21]).

### 2.3. Comparison of the SARS-CoV-2 Epidemic in Different Countries

**Figure 2:**
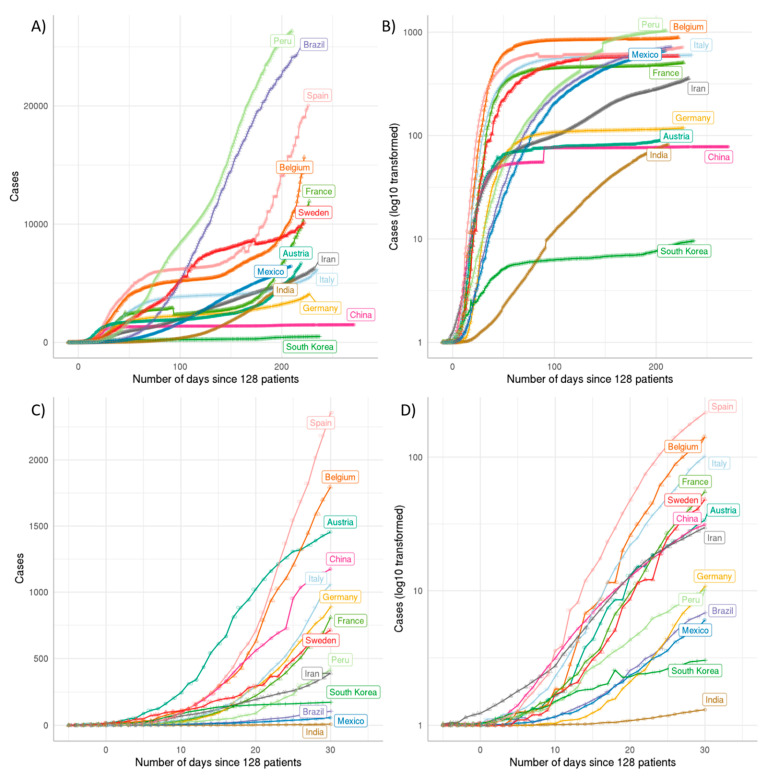
Cumulative cases per million population. (**A**) Cumulative patient numbers divided by the population in million illustrate the respective epidemic situation. (**B**) Cumulative death numbers should avoid biases coming from different diagnostic capabilities and standards. (**C**) Cumulative patient cases per million population up to day 30, (**D**) Cumulative death cases per million population up to day 30. For China the data of Hubei province were analyzed as here full data were available and infection spread was successfully nearly completely limited to this region. The figure was generated by us using the data obtained from RKI/Tencent with a R/nCov2019 script [22].

The cumulative data from different countries were collected and sorted according to patient cases and fatalities. All data on the pandemic were further normalized to the population of each country. When standardized to a common threshold the curves reveal for most of the countries an exponential increase of cases with comparable growth rates during the first 15 days of infection spread after the threshold time point. We used a time point as the threshold when exactly 128 (2^7^) COVID-19 patients were observed in each country. An exception to this common pattern can be seen in South Korea with a much lower slope of growth of identified cases. We speculate that this is due to better protection from the beginning of the pandemic because wearing a protective mask is widespread in the Korean population even before the pandemic. Countries such as Colombia, South Africa, Italy, but also Belgium and Mexico, have much higher disease burden (Figure 2A) and death toll (Figure 2B) than countries like South Korea, compared to their population size. The area between 0 and 30 days are shown in Figure 2C,D.

**Figure 3:**
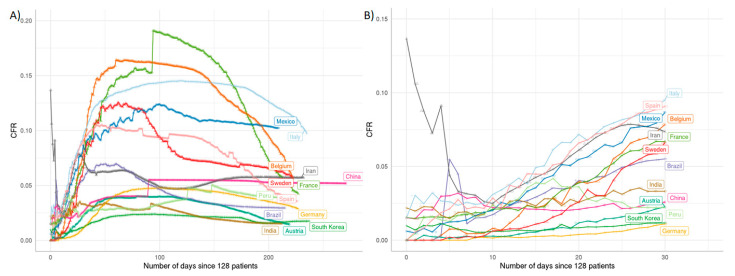
Case fatality rate (CFR) comparison of SARS-CoV-2 infection over time. The CFR values over time are illustrated by the curves, specific colors indicate individual countries as labeled in the same color. (**A**): CFR over time for individual countries until October 2020. The dramatic rise of most curves after the early phase indicates partly that the public health system of a country may have reached its maximum limit of treatment capability. The dropping tendency of each curve indicates the public health system keeps recovering from the early crisis. (**B**): enlarged view up to day 30 allows close inspection of the early phase of infection spread. The figure is de novo generated using the public data from RKI/Tencent, processed by the R/nCov2019 script [22].

In pandemic outbreaks, we usually observe a declining case fatality rate (CFR) over time due to an underestimation of cases at the beginning of an epidemic. To analyze whether we see the same trend with COVID-19, we plotted the CFR against time (Figure 3). Surprisingly, we did not observe a decline of CFR over time for most of the countries but instead a steep increase. This steep increase is most prominently seen in Italy, Mexico and Belgium. The reasons for this strong increase in CFR over time are probably related to reaching the capacity limits of the health system in these countries due to overshooting numbers of COVID-19 cases. In contrast, other countries such as Germany, South Korea and Finland show a relatively stable development over time indicating a good control of the COVID-19 infection. The difference in the case fatality rate between these countries that have managed to control the infection probably reflects different numbers in testing. For instance, Germany and South Korea did perform many tests, including the testing of patients with mild symptoms.

### 2.4. Poor MHCII Coverage Correlates with High Case Fatality Rate

**Figure 4:**
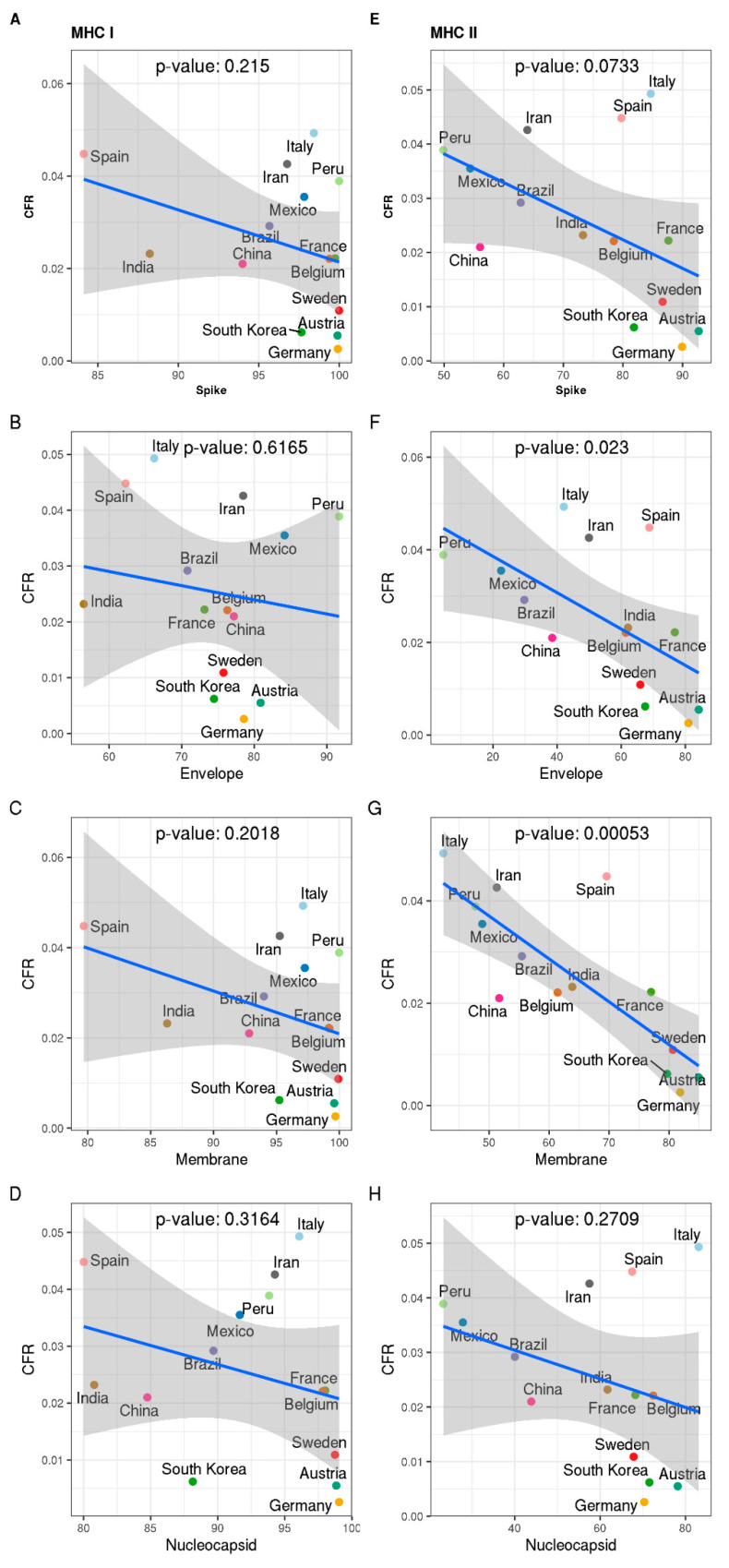
Correlation of predicted MHC-presentation and case fatality rate (CFR) in different countries. Predicted MHC presentations (left: MHC I; right: MHC II) for different structural proteins of SARS-CoV-2 were correlated with the CFR reported for each country at day 15 after the first 128 cases. The figures include a *p*-value as calculated for linear regression (by ggpubr package). The shaded areas indicate two standard deviations (confidence intervals) around the linear correlation. Predicted MHC-presentation of different SARS-CoV-2-proteins for different populations and case fatality rate (CFR) observed in different countries reveals a strong negative correlation for predicted MHCII-presentation of SARS-CoV-2-epitopes and CFR for the membrane protein (*p*-value: 0.00053, **G**), for Envelope protein (*p*-value: 0.023, **F**) and, only clear (*p* < 0.1) but no longer strong for the Spike protein (*p* = 0.0733) (**E**), but not for MHCI (membrane protein: panel (**C**), envelope protein: (**B**), spike protein: (**A**), nucleocapsid protein: (**D**) or nucleocapsid protein (**H**). Good MHCII presentation is an important prerequisite for T-cell-dependent antibody production. Supplementary Figure S4 shows that these strong correlations change into just a trend at day 30 (after the first 128 cases) and decay completely with even more time (Table S8 compares correlations for different SARS-CoV-2-epitopes and CFR at different time points). The detected strong correlations suggest that predicted differences in the adaptive immune response in different populations may play an important role in the observed clear differences of case fatality rates observed in different countries for this first phase of infection spread. A strong adaptive response directed at the spike, envelope and particularly the membrane protein seems important to prevent further spread of SARS-CoV-2-infection. Specific controls include results and tool validation on other viruses (Table S6) as well as results for scrambled sequences (Table S7).

We correlated the overall predicted MHC-presentation of the four structural SARS-CoV-2-proteins for different populations with the case fatality rate (CFR) observed in different countries at day 15 since 128 patients (Figure 4; CFR data taken from Figure 2). We chose this time point for several reasons: (a) at this time point, significant differences in the CFR between different countries can already be observed; (b) secondary effects that may also impact CFR, like (i) overwhelming the health system capacity as observed in some countries due to overshooting numbers of patients, (ii) varying strain heterogeneity (see, e.g., current SARS-CoV-2 variation at https://nextstrain.org/ncov/global; 4992 genomes sampled December 2019 till the end of September 2020 according to Nextstrain database [23]—accessed 15 October 2020) and (iii) different protective measures are likely not yet compromising the SARS-CoV-2 associated CFR at this comparatively early time point. This confirms that at later time points, a very significant correlation by day 15 of infection spread (day 0 is the first 128 cases observed) becomes only a trend (day 30, 100; Table S8), which disappears on later days. However, in this early phase (to day 15), we observed a statistically significant negative correlation between the CFR observed in different countries and the predicted MHCII coverage for populations in these countries for the membrane protein (Figure 4G, *p*-value: 0.00053) and the envelope protein (Figure 4F, *p*-value: 0.023), but not for other proteins or MHCI coverage.

The quality of MHCII presentation by the T-cell is an important prerequisite for T-cell-dependent antibody production. Besides the envelope protein and membrane protein, the SARS-CoV-2 surface (spike) protein also shows a clear correlation but only below the *p*-value: 0.1 level (Figure 4E, *p*-value: 0.0733), since it is probably the important target for neutralizing antibodies and it is, therefore, a remarkable observation that predicted MHCII-coverage of epitopes of this protein reveals the strongest correlation with the observed CFR in different countries.

## 3. Discussion

The correlations in this study investigate differences in the adaptive immune response to SARS-CoV-2 envelope proteins in different populations. Several observed strong correlations suggest that these may play a role in the observed clear variation in fatality rates in different countries. According to these observations, strong MHC-restricted responses directed at the membrane protein and the envelope protein would be important to achieve better control over SARS-CoV-2-infection and are implied to lower CFR. The correlation of MHCII-presentation of the predicted epitopes of membrane, envelope and spike protein with a low CFR observed in different populations supports also the importance of these proteins as vaccine and potential therapeutic candidates.

As a major conclusion of our study, we see that overall the strong epitope binders in T-cells are underrepresented in the Italian population as compared to the German population. However, the current data are not strong enough to identify particular alleles that definitely drive the differences. What we did study and deliver in our work are the differences in adaptive immune response regarding T-cell and B-cell epitope presentation averaging on the global population, not looking at individual populations but covering most of the prevalent alleles in most of the world’s major geographic regions. We primarily investigated MHC-restricted responses, which comprise both, humoral and cell-mediated reactions.

Doubtless, many other factors may also contribute to country-specific differences: for instance in some Asian countries such as in Japan may be connected to cultural habits such as many people wearing a face mask even before the pandemic. Nevertheless, for viral diseases such as H7N9 influenza A virus, analysis of T-cell immunity in human populations applying refined immune-informatics provided also here valuable insights such as clear ethnic differences [24,25] and a basis for suitable vaccine strategies including even pandemic preparedness [26].

Regarding responses in specific subpopulations, the panel and the predictive power are not strong enough. For example, alleles associated with the DR14 and DR16 serological families, or various DR4 splits such as DR0404 or DR0407 (which will have different binding patterns than DR0401) that may be prevalent in the “negative” populations with reasonably high frequency, but not in the “positive” populations, are not represented in our prediction analysis. Associations of infectious diseases with the HLA DR4 type of the host have been found among distinct populations, racial or ethnic groups. In Mexico, a greater frequency of HLA-DR4 antigens was found in patients with Chagas’ disease with an increased frequency of HLA-DR16 antigens compared to asymptomatic patients or healthy controls [27]. Particularly HLA-DR4 and its splits DR0404 and DR0407, which are highly prevalent types in the Mayos ethnic group of Mexico, have been associated with susceptibility to infectious diseases and inflammatory disease. The exalted inflammatory response in HLA-DR4 carriers may account for the high fatality rates due to COVID-19 in the Mexican population. Moreover, only if we really sample population-specific high abundant but otherwise rare alleles, for instance in Peru, could it not be that such a population is better protected than we estimate? However, as soon as there would be such protection by a certain allele combination in this “negative” population, it would be reflected by a lower case fatality rate for that specific population even if the representation of alleles from the selected most frequent HLA molecules has poor coverage. However, we noticed a strong correlation exactly and only with the most prevalent alleles. At least on the global correlation, any additional rare or population-specific alleles and their potential protective value did not alter the strong correlation we observed.

Another important fact is the impact of COVID-19 on different races and ethnicities, which was noticed in several studies [28,29,30]. A study by Rossen et al. compared the death dates in various ethnicities during 10 months of 2020 in the USA. They observed an increased death rate compared with previous years in each of the monitored populations. The highest mortality was observed in the Hispanic community (53.6% above average), followed by Asian persons (36.6%), Black persons (34.6%) and American Indian/Alaska Native (28.9%). The lowest increase in mortality was found in the White non-Hispanic population, which was 11% higher compared to previous years [31]. Comparably, we focused on the HLA allele’s ability to bind the structural protein of SARS-CoV-2. In Figure S2, we present similar results compared to the works mentioned above. For the MHCI class, the binding abilities were fairly even, however, for the MHCII, they varied considerably. The highest binding was detected in Caucasoid (White) population, followed by the Asian, mixed, Polynesian, Hispanic and Black population. The lowest affinity was observed by Native Americans and Indians (Figure S2). However, it must be emphasized that in addition to the genetic predisposition, there are other factors such as sharing apartments, facilities or communal areas, economic status, the prevalence of diseases such as diabetes and cardiovascular diseases, availability of health care and types of jobs (reviewed in [32])

There is also another important point to discuss: could it be that other factors (e.g., health systems, non-HLA immunological factors) have an equally high correlation or even higher? Well, firstly we only observe a correlation, not a molecular proof. To claim molecular proof, we would need to consider various additional factors: rare antigens, highly represented antigens in local populations, and importantly, direct molecular assays of the immune response. Instead, we focused here only on the correlation of MHC epitopes. However, this correlation is surprisingly strong for the membrane protein (*p*-value: 0.00053) and for the envelope protein (*p*-value: 0.023), and still clear for the viral spike protein (*p* < 0.1; i.e., *p*-value: 0.0733 for linear regression). However, we cannot, of course, rule out that a completely different factor such as health system and non-HLA immunological factors could bear out an even higher correlation. However, for the latter, we did all reasonable efforts to account for this by (i) purposely averaging on the world population so that local trends do not lead to biases (but then of course in local regions things may be different), (ii) monitoring overall immune response by looking at case fatality rate and (iii) by avoiding multiple other comparisons and hypothesis as then the statistical correlation has to be corrected and is weakened by multiple testing. In addition, the obviously higher correlation for MHCII compared to MHCI may indicate that CD4 cells play a more crucial role in fighting against the SARS-CoV-2 infection for the human immune system, in particular for severe COVID-19 patients. This may lead us to reconsider the strategy and development of novel therapeutics. An exception is a nucleocapsid, though highly expressed, this is unlikely able to offer sufficient neutralizing antibody response on its own.

We report a correlation of MHCII stimulatory epitopes in different populations with CFR for COVID-19 patients. It is overall strong and solid on a global level and present for the early phase of the infection spread. Analysis of subpopulations could reveal specific local immunity to SARS-CoV-2. Unfortunately, such effects are comparatively small on the level of a global analysis done here as otherwise, the overall correlation with global well-represented antigens would have been much more diluted. However, we hope that our study will be an incentive to also hunt down and in fact, judge other factors influencing and correlating with CFR such as health system or for instance pandemic control measures. We think these factors should be important as we see that after some time the strong correlation with naïve immunity and epitope representation breaks down and so other factors clearly then take over controlling the SARS-CoV-2 spread. Moreover, such other factors, in particular, health system status and different control measures are instrumental to control the SARS-CoV-2 pandemic further.

## 4. Methods

### 4.1. Dataset Collection

The protein sequences of SARS-CoV-2 were obtained from the NCBI (GenBank accession number: MN908947.3; severe acute respiratory syndrome coronavirus 2 isolate Wuhan-Hu-1). Accession numbers, annotations, PDB identifiers and the length of analyzed proteins are given in Table 3. Protein domain mapping was performed using the Pfam database [33] (Table S1).

### 4.2. Prediction of Linear B-Cell Epitopes

Protein sequences of SARS-CoV-2 were screened for linear continuous B-cell epitopes using three tools:

BepiPred Linear Epitope Prediction tool (ver. 2) (http://www.cbs.dtu.dk/services/BepiPred, accessed date: 5 February 2020 [34]) was used to find the epitopes using the random forest algorithm to identify the epitopes annotated from antigen–antibody-protein structures with ≥0.5 and longer than 9 amino acids.

IEDB Database [35] used the Koloskar and Tongaonkar antigenicity method with a threshold value of 1.05 and epitope length longer than 9 [36]. Validated epitopes are listed in the database IEDB (https://www.iedb.org, accessed date: 5 February 2020).

The parameters for the ABCpred Tool (http://crdd.osdd.net/raghava/abcpred, accessed date: 5 February 2020) [37]) were used as follows: threshold 0.51 and epitope length of 10 amino acids. An overlap filter was applied.

### 4.3. Prediction of T-Cell Epitopes

T-cell epitopes prediction was performed using TepiTool (http://tools.iedb.org/tepitool; accessed date: 10 February 2020 [38]). To predict MHCI binders, we used the set of 27 most frequent alleles (Table 4) and NetMHCpan method was used to assess the best binding 9mer. The selection criterion was a cut-off of IC50 ≤ 500. The MHCII alleles were predicted with epitope length 12–18 amino acid and IC50 ≤ 1000. The set of used alleles is listed in Table 4. Moreover, each SARS-CoV-2 protein was analyzed by VaxiJen v2.0 antigen prediction server using the default parameters (threshold >0.4) to analyze the antigenicity of full-length viral proteins. [39].

### 4.4. Population Coverage

The distribution of COVID-19 recognizing MHCI and MHCII alleles was assessed using the IEDB population coverage tool [31]. Here, we focused only on structural proteins (nucleocapsid phosphoprotein, surface/spike glycoprotein, membrane protein and envelope protein). We first filtered epitopes with high binding affinity (IC < 100 nM). Additionally, for the MHCII, we selected only epitopes recognized by two and more alleles, except for the envelope protein, due to the low number of epitopes. Population coverage was calculated using default parameters separately for each group of alleles. A list of selected epitopes and recognizing alleles is given in the supplement (Table S4). For analysis, we included all available countries and ethnicities listed in Allele Frequency Net Database [40].

### 4.5. COVID-19 Spread Analysis

The analysis was implemented in R by acquiring data from the nCov2019 library [22] and the presentation of the data was done using the ggplot2 library [41]. Daily data of selected countries, (Germany, Italy, Spain, India, Peru, Mexico, France, China, South Korea, Belgium, Sweden, Iran, Austria and Brazil), were processed and compared, we further aligned the log-transformed curves upon the time point when the cumulative patient cases reached 128 (27) to compare the public health systems among different countries, including both patient case curves and death case curves. The next analysis step is to investigate the development of the epidemic according to the population of each country. For China, there were only full data for the population of Hubei province available and hence considered. However, particularly for the early phase of infection spread this should not cause a major skew of the data as China has successfully limited the epidemic region mostly to this province. Case fatality rate (CFR) was inferred by calculating the ratio of cumulative death numbers divided by cumulative patient cases. The curves were also aligned to the time point when the patient number reaches 128.

### 4.6. Correlation Analysis

During the outbreak, the proportion of death from COVID-19 (CFR) is observed for different countries at 15 days since 128 cases were reported. Accordingly, antigen presentation is collected as a percentage of MHCI and MHCII epitopes. The linear correlation is calculated using the “ggpubr” package in R, and a Pearson method is applied to run the test. We hence assume that the population data are normally distributed.

## 5. Conclusions

We hypothesize that countries with poor case fatality rates are associated with poor immune response. We predict immunity by monitoring well-represented epitopes, whether these are already sufficient to derive a good correlation with the observed immunity, which is given inversely correlating parameter case fatality rate. We observe that this is really the case in the initial phase of infection. Our analysis combines multiple approaches to investigate the pandemic of SARS-CoV-2 in different countries. There are striking differences predicted for natural population immunity in different populations. Differences in the severity of COVID-19 disease observed in different appear to be related to differences in the potential of different populations to present SARS-CoV-2 epitopes on MHCII in a naïve population (up to day 15 of infection spread; starting from 128 observed cases). Geographical areas with a high incidence of virus and disease burden such as Italy and Iran have a low epitope-binding MHCII-repertoire compared to countries with less severe disease progression such as Germany, Austria, Sweden, Norway and South Korea. We emphasize here that these are only correlations but these were confirmed using well-established epitope prediction tools that include experimental validation data and predict successfully also established SARS-CoV-2 and other viral epitopes. Moreover, the case fatality rate is a direct indicator of whether the immune response in the population is sufficient, including other epitopes or other immune factors. Our results highlight the importance of the SARS-CoV-2 structural proteins as a target to gain MHCII restricted control over the infection, e.g., by vaccine strategies and should encourage further studies probing the molecular immunology of SARS-CoV-2 further and examining MHCII alleles as potential individual risk factors in COVID-19. Future studies should focus on specific geographic regions as well as other factors taking over after the initial phase (later than day 15 after reaching 128 observed cases) such as health system, potential strain variation effects and, most importantly, different control measures. The evidence presented here suggests that epitopes predicted to provide broad coverage worldwide are also likely to provide broad coverage in specific populations and correlated strongly and inversely with a case fatality rate in the early phase of infection spread.

## Figures and Tables

**Table 1:** Comparison of the number of the predicted epitopes for each SARS-CoV-2 protein.

	B Cell Epitope (No. of Epitopes)			T Cell Epitope (No. of Epitopes) *		VaxiJen v2.0 Score
Protein Name	IEDB	BepiPred-2.0	ABCpred	TepiTool: MHC Class I	TepiTool: MHC Class II	
Orf1ab polyprotein	228	157	677	47894789	10051	0.4624
Surface glycoprotein	115	28	117	766765	1751	0.4646
ORF3a protein	11	6	24	224	64372	0.4945
Envelope protein	1	1	5	3886	19119	0.6025
Membrane glycoprotein	6	3	20	225224	379	0.5102
ORF6 protein	1	1	6	2054	2591	0.6131
ORF7a protein	4	42	11	438989	167	0.6441
ORF8 protein	42	43	11	245757	15134134	0.6502
Nucleocapsid phosphoprotein	0	7	38	68152152	42311311	0.5059
ORF10 protein	1	0	3	214949	59	0.7185
Sum of predicted epitopess	409	288	912	6491	13434	

* for T-cells, the number of unique epitope/Major Histocompatibility Complex (MHC) combinations is listed.

**Table 2:** Epitopes of structural proteins for each Human Leukocyte Antigen (HLA) allele *^.^

MHC Class I					MHC Class II				
Allele	Spike Protein	Envelope Protein	Membrane Protein	Nucleo Capsid Protein	Allele	Spike Protein	Envelope Protein	Membrane Protein	Nucleo-Capsid Protein
HLA-A*01:01	6	1	3	1	HLA-DPA1*01:03/DPB1*02:01	73	8	22	8
HLA-A*02:01	36	11	17	2	HLA-DPA1*02:01/DPB1*01:01	70	5	21	10
HLA-A*02:03	75	13	19	7	HLA-DPA1*02:01/DPB1*05:01	22	2	9	1
HLA-A*02:06	74	13	19	11	HLA-DPA1*03:01/DPB1*04:02	46	4	14	4
HLA-A*03:01	22	2	4	5	HLA-DQA1*01:01/DQB1*05:01	20	0	7	2
HLA-A*11:01	41	2	9	9	HLA-DQA1*01:02/DQB1*06:02	71	5	14	15
HLA-A*23:01	25	2	11	3	HLA-DQA1*03:01/DQB1*03:02	7	0	1	2
HLA-A*24:02	19	0	8	2	HLA-DQA1*04:01/DQB1*04:02	9	0	2	3
HLA-A*26:01	8	1	2	2	HLA-DQA1*05:01/DQB1*02:01	32	0	4	5
HLA-A*30:01	53	5	19	24	HLA-DQA1*05:01/DQB1*03:01	82	2	11	31
HLA-A*30:02	25	3	8	4	HLA-DRB1*01:01	158	10	27	30
HLA-A*31:01	34	4	14	14	HLA-DRB1*03:01	37	2	8	5
HLA-A*32:01	26	4	9	6	HLA-DRB1*04:01	106	6	18	21
HLA-A*33:01	17	1	11	5	HLA-DRB1*04:05	101	6	20	20
HLA-A*68:01	49	3	13	11	HLA-DRB1*07:01	123	9	22	14
HLA-A*68:02	57	8	11	8	HLA-DRB1*08:02	68	6	18	14
HLA-B*07:02	10	0	3	5	HLA-DRB1*09:01	124	7	22	22
HLA-B*08:01	15	1	3	4	HLA-DRB1*11:01	77	5	19	15
HLA-B*15:01	48	5	11	7	HLA-DRB1*12:01	46	6	18	6
HLA-B*35:01	42	5	5	8	HLA-DRB1*13:02	103	8	20	15
HLA-B*40:01	9	1	1	0	HLA-DRB1*15:01	98	8	23	17
HLA-B*44:02	6	0	1	1	HLA-DRB3*01:01	41	2	6	4
HLA-B*44:03	6	0	1	1	HLA-DRB3*02:02	53	5	9	8
HLA-B*51:01	3	0	1	1	HLA-DRB4*01:01	93	8	24	17
HLA-B*53:01	16	0	6	4	HLA-DRB5*01:01	91	5	20	22
HLA-B*57:01	13	0	5	3					
HLA-B*58:01	30	1	10	4					
Sum	765	86	224	152		1751	119	379	311

* Summary numbers are given for each allele. The detailed information about the binding capacity and epitope position is listed in Table S3**.**

**Table 3:** SARS-CoV-2 proteins used in the immunogenic analysis.

Accession No.	Protein Annotation	PDB	Length (aa)
QHD43415.1	orf1ab Polyprotein orf1ab	7COM	7096
QHD43416.1	surface/Spike glycoprotein	6X79	1273
QHD43417.1	Protein ORF3a	6XDC	275
QHD43418.1	Envelope small membrane protein (E)	not available	75
QHD43419.1	Membrane glycoprotein (M)	not available	222
QHD43420.1	Non-structural protein 6	not available	61
QHD43421.1	Protein 7a	6W37	121
QHD43422.1	Non-structural protein 8	7JX6	121
QHD43423.2	Nucleocapsid phosphoprotein (NC)	6ZCO	419
QHI42199.1	ORF10 protein	not available	38

**Table 4:** Alleles used in the TepiTool predictions.

Allele Class	Alleles (Human)
MHC Class I	A*01:01, A*02:01, A*02:03, A*02:06, A*03:01, A*11:01, A*23:01, A*24:02, A*26:01, A*30:01, A*30:02, A*31:01, A*32:01, A*33:01, A*68:01, A*68:02, B*07:02, B*08:01, B*15:01, B*35:01, B*40:01, B*44:02, B*44:03, B*51:01, B*53:01, B*57:01, B*58:01
MHC Class II	DRB1*01:01, DRB1*03:01, DRB1*04:01, DRB1*04:05, DRB1*07:01, DRB1*08:02, DRB1*09:01, DRB1*11:01, DRB1*12:01, DRB1*13:02, DRB1*15:01, DRB3*01:01, DRB3*02:02, DRB4*01:01, DRB5*01:01, DPA1*01/DPB1*04:01, DPA1*01:03/DPB1*02:01, DPA1*02:01/DPB1*01:01, DPA1*02:01/DPB1*05:01, DPA1*03:01/DPB1*04:02, DQA1*01:01/DQB1*05:01, DQA1*01:02/DQB1*06:02, DQA1*03:01/DQB1*03:02, DQA1*04:01/DQB1*04:02, DQA1*05:01/DQB1*02:01, DQA1*05:01/DQB1*03:01

* Summary numbers are given for each allele.

## Data Availability

All data used for this study are available from the manuscript, its supplements and its figures. For databases used the link and time of access are given.

## Supplementary Materials

The following are available online at https://www.mdpi.com/1422-0067/22/5/2630/s1, Table S1. Domain mapping of SARS-CoV-2 proteins; Table S2. Prediction of B-cells epitopes; Table S3. Prediction of T-cells epitopes; Table S4. List of most frequent MHC class I epitopes (predicted in silico); Table S5. Population coverage analysis based on most frequent T-cell epitopes (predicted in silico); Table S6. Comparison of SARS-CoV, SARS-CoV-2 and MERS epitopes; Table S7. The dataset of randomized sequenced and generated population coverage analysis for each MHC class I and II alleles. Table S8. The comparison of correlations for different SARS-CoV-2-epitopes and CFR at different time points. Figure S1. Comparison of B-cell sand T-cells epitopes in the previous SARS virus (strain TW3) and SARS-CoV-2 represented on a surface (spike) protein and membrane protein. Experimentally validated epitopes are taken from [42].; Figure S2. Population coverage analysis of most frequent T-cells epitopes (predicted in silico) for different ethnical groups in the United States. Panel A—MCHI, Panel B—MHCII, Panel C—percentage representation of ethnicity according to the Allele Frequency Net Database for US population.

## Abbreviations

CFR	Case Fatality Rate
SARS	Severe Acute Respiratory Syndrome
MERS	Middle East Respiratory Syndrome
MHC	Major Histocompatibility Complex
HLA	Human Leukocyte Antigen
