# Supplementary material for: Population-Predicted MHC Class II Epitope Presentation of SARS-CoV-2 Structural Proteins Correlates to the Case Fatality Rates of COVID-19 in Different Countries
Source: Int J Mol Sci. 2021 Mar 5;22(5):2630. doi: 10.3390/ijms22052630 (PMC7961590; doi:10.3390/ijms22052630)
Supplement: Supplementary file 1 [file ijms-22-02630-s001.zip › Fig.S2.pdf]

C

Ethnic	Distribution according Allele Frequency Net Database
Black	13,708%
Amerindian	0,941%
Asian	2,861%
Caucasoid	55,973%
Hispanic	14,862%
Mestizo	0,129%
Mixed	5,466%
Oriental	4,151%
Australonesian	0,770%
Polynesian	0,174%
Arab	1,076%

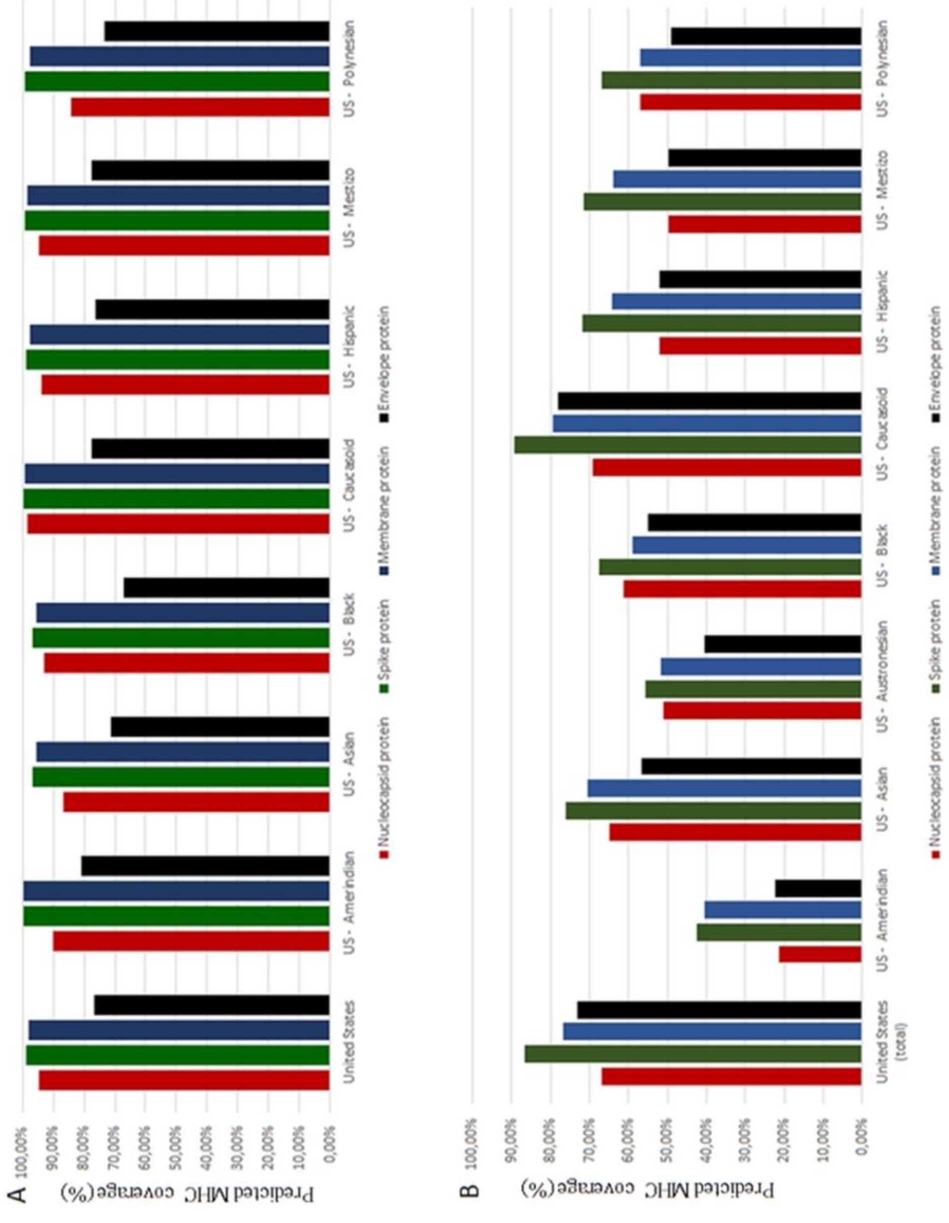

Analyzing predicted MHC II coverage according to ethnicity for the United States (Fig. S2).
Population coverage analysis of most frequent T-cells epitopes (predicted *in silico*) in selected ethnical groups in the United States. Panel A – MCHI, Panel B – MHCII, Panel C – percentage representation of ethnicity according to the public Allele Frequency Net Database for US population. Given are the predicted coverages for nucleocapsid protein, spike protein, membrane and envelope protein. The coverages are varying for different ethnicities. This includes United States caucasian population. Here the exact figures are 69,05%, 89,12%, 79,43% and 78,04%, respectively, while for the whole US population the figures four these four proteins are 66,71%, 86,68%, 76,89% and 73,26%, respectively. The non-hispanic white population in USA is 56% of the total population (see panel C).
