# Supplementary material for: Population-Predicted MHC Class II Epitope Presentation of SARS-CoV-2 Structural Proteins Correlates to the Case Fatality Rates of COVID-19 in Different Countries
Source: Int J Mol Sci. 2021 Mar 5;22(5):2630. doi: 10.3390/ijms22052630 (PMC7961590; doi:10.3390/ijms22052630)
Supplement: Supplementary file 1 [file ijms-22-02630-s001.zip › TableS1_Domain.docx]

**Table S1. Domain analysis result of SARS-CoV-2 proteins**

| **Protein (NCBI ID)** | **Family (Pfam accession number)** | **Position** | **Bit score** | **E-value** |
| --- | --- | --- | --- | --- |
| **ORF1ab polyprotein (QHD43415.1)** | NSP11 (PF06471) | 5929-6520 | 1026.4 | 3.3e-309 |
|  | NSP16 (PF06460) | 6800-7095 | 546.4 | 1.4e-164 |
|  | Peptidase_C30 (PF05409) | 3292 - 3582 | 448.1 | 1.2e-134 |
|  | Viral_protease (PF08715) | 1564-3882 | 434.2 | 3.1e-130 |
|  | Corona_RPol_N (PF06478) | 4406-4626 | 361.7 | 3.7e-108 |
|  | nsp8 (PF08717) | 3943-4140 | 317.3 | 4.0e-95 |
|  | SUD-M (PF11633) | 1351-1493 | 256.7 | 4.0e-77 |
|  | Corona_RPol_N (PF06478) | 4627-4758 | 236.8 | 3.4e-70 |
|  | Nsp1 (PF11501) | 13-127 | 213.4 | 5.5e-64 |
|  | NSP10 (PF09401) | 4262-4384 | 199.2 | 1.7e-59 |
|  | nsp9 (PF08710) | 4141-4253 | 160.8 | 1.3e-47 |
|  | Corona_NSP4_C (PF16348) | 3166-3262 | 135.9 | 6.3e-40 |
|  | NAR (PF16251) | 1909-2019 | 128.1 | 2.6e-37 |
|  | nsp7 (PF08716) | 3860-3942 | 123.2 | 4.8e-36 |
|  | Nsp3_PL2pro (PF12124) | 1497-1561 | 103.9 | 2.7e-30 |
|  | DUF3655 (DUF3655) | 920 - 987 | 80.4 | 8.5e-23 |
|  | Macro (PF01661) | 1058-1165 | 61.3 | 8.7e-17 |
|  | AAA_30 (PF13604) | 5599-5731 | 29.7 | 4.8e-07 |
|  | AAA_12 (PF13087) | 5812-5899 | 27.3 | 2.5e-06 |
| **S protein** | Corona_S2 (PF01601) | 671-1270 | 885.9 | 1.4e-266 |
|  | Spike_rec_bind (PF09408) | 330-583 | 251.5 | 6.6e-75 |
| **ORF3A** | APA3_viroporin (PF11289) | 1-274 | 474.0 | 1.5e-142 |
| **E** | NS3_envE (PF02723) | 1-74 | 32.2 | 8.2e-08 |
| **M** | Corona_M (PF01635) | 4-221 | 318.8 | 1.4e-95 |
| **ORF6** | Sars6 (PF12133) | 1-61 | 104.4 | 2.1e-30 |
| **ORF7a** | SARS_X4 (PF08779) | 16-98 | 178.0 | 2.9e-53 |
| **ORF8** | Corona_NS8 (PF12093) | 1-118 | 150.0 | 3.8e-44 |
| **N** | Corona_nucleoca (PF00937) | 14-377 | 547.6 | 1.2e-164 |

The domain analysis was performed by using the Pfam database.

The **polyprotein ORF1ab** exhibits 19 domains which are characteristic for *Coronaviridae*. Moreover, we found also two motifs in the Prosite database: CPSASE_2 (Carbamoyl-phosphate synthase subdomain, PS00867) at position 2061-2068 and Lipocalin (PS00213) at position 4982-4993. S protein contains Corona S2 glycoprotein family and spike receptor-binding domain, which are crucial for viral entry to the host cell.

**Protein E** possesses the NS3_envE domain (non-structural protein NS3/Small envelope protein E). This domain is involved in multiple functions of viral life, including budding, envelope formation and assembly of viral particles^1^. The APA3_viroporin family, which is located in the N-terminus of the ORF3 protein, plays probably a role in the release of the virus from infected cells as well as in viral assembly^2^.

The **Corona_M** (coronavirus M matrix/glycoprotein) family is located on the structural membrane protein and it is involved in formation of envelope ^3^.

**Domain Sars6** located in ORF6 and Corona_NS8 (ORF8) has not yet a known function. The **Sars_X4 domain** (ORF7a protein) is involved probably in the replication of the virus and incudes apoptosis in the caspase-dependent pathway ^4,5^. Moreover, our analysis showed the presence of several epitopes in the protein domains:

|  | **B-cell epitopes (Nr.)** | | | **T-cell epitopres (Nr.)** | |
| --- | --- | --- | --- | --- | --- |
| **Protein/Domain** | **IEDB** | **BepiPred** | **ABCpred** | **MHC I** | **MHC II** |
| S (surface protein)/ Spike_rec_bind | 5 | 7 | 42 | 68 | 67 |
| S protein/ Corona_S2 | 12 | 11 | 23 | 69 | 89 |
| E/NS3_envE | 1 | 1 | 5 | 30 | 11 |
| M/Corona_M | 6 | 3 | 20 | 84 | 31 |
| N/Corona_nucleoca | 3 | 8 | 37 | 68 | 45 |

1 Schoeman, D. & Fielding, B. C. Coronavirus envelope protein: current knowledge. *Virol J* **16**,

69, doi:10.1186/s12985-019-1182-0 (2019).

2 Shen, S. *et al.* The severe acute respiratory syndrome coronavirus 3a is a novel structural protein. *Biochem Bioph Res Co* **330**, 286-292, doi:10.1016/j.bbrc.2005.02.153 (2005).

3 Armstrong, J., Niemann, H., Smeekens, S., Rottier, P. & Warren, G. Sequence and Topology of a Model Intracellular Membrane-Protein, E1-Glycoprotein, from a Coronavirus. *Nature* **308**, 751-752, doi:DOI 10.1038/308751a0 (1984).

4 Taylor, J. K. *et al.* Severe Acute Respiratory Syndrome Coronavirus ORF7a Inhibits Bone Marrow Stromal Antigen 2 Virion Tethering through a Novel Mechanism of Glycosylation Interference. *J Virol* **89**, 11820-11833, doi:10.1128/Jvi.02274-15 (2015).

5 Schaecher, S. R., Touchette, E., Schriewer, J., Buller, R. M. & Pekosz, A. Severe acute respiratory syndrome coronavirus gene 7 products contribute to virus-induced apoptosis. *J Virol* **81**, 11054-11068, doi:10.1128/Jvi.01266-07 (2007).
