# Supplementary material for: Population-Predicted MHC Class II Epitope Presentation of SARS-CoV-2 Structural Proteins Correlates to the Case Fatality Rates of COVID-19 in Different Countries
Source: Int J Mol Sci. 2021 Mar 5;22(5):2630. doi: 10.3390/ijms22052630 (PMC7961590; doi:10.3390/ijms22052630)
Supplement: Supplementary file 1 [file ijms-22-02630-s001.zip › TableS4_Epitope_population.docx]

| **Supp. Table 4a: MHC class II epitopes and their recognizing alleles used for the population coverage analysis.** | | |
| --- | --- | --- |
| Spike protein | AAEIRASANLAATKM | HLA-DRB1*01:01  HLA-DRB1*13:02  HLA-DRB3*02:02  HLA-DRB1*04:01  HLA-DQA1*05:01/DQB1*03:01  HLA-DRB1*07:01  HLA-DRB1*07:01  HLA-DRB1*09:01 |
|  | AQKFNGLTVLPPLLT | HLA-DRB1*01:01  HLA-DRB1*04:05  HLA-DRB1*04:01  HLA-DRB1*09:01  HLA-DRB1*07:01 |
|  | ATRFASVYAWNRKRI | HLA-DRB5*01:01  HLA-DRB1*01:01  HLA-DRB1*07:01  HLA-DRB1*09:01  HLA-DRB1*11:01  HLA-DRB1*15:01 |
|  | DEMIAQYTSALLAGT | HLA-DRB1*01:01  HLA-DRB1*15:01 |
|  | DLFLPFFSNVTWFHA | HLA-DRB1*01:01  HLA-DRB1*15:01  HLA-DRB1*07:01  HLA-DRB1*04:05  HLA-DPA1*01:03/DPB1*02:01  HLA-DRB1*04:01  HLA-DRB1*09:01  HLA-DRB3*02:02 |
|  | EDLLFNKVTLADAGF | HLA-DRB1*13:02  HLA-DRB1*01:01  HLA-DRB1*13:02 |
|  | FFSNVTWFHAIHVSG | HLA-DRB1*07:01  HLA-DRB1*01:01  HLA-DRB1*15:01  HLA-DRB1*09:01 |
|  | FGEVFNATRFASVYA | HLA-DPA1*01:03/DPB1*02:01  HLA-DRB1*07:01  HLA-PA1*02:01/DPB1*01:01  HLA-DRB1*01:01  HLA-DRB1*09:01  HLA-DPA1*03:01/DPB1*04:02  HLA-DRB1*11:01 |
|  | FSTFKCYGVSPTKLN | HLA-DRB1*01:01  HLA-DRB1*07:01 |
|  | GSNVFQTRAGCLIGA | HLA-DRB1*01:01  HLA-DRB1*07:01 |
|  | GWTFGAGAALQIPFA | HLA-DQA1*05:01/DQB1*03:01  HLA-DRB1*09:01  HLA-DRB1*07:01 |
|  | IIAYTMSLGAENSVA | HLA-DRB1*01:01  HLA-DRB1*09:01  HLA-DRB1*07:01  HLA-DRB1*04:01  HLA-DRB5*01:01  HLA-DRB1*04:05 |
|  | ITRFQTLLALHRSYL | HLA-DRB5*01:01  HLA-DRB1*01:01  HLA-DRB1*11:01  HLA-DRB1*15:01  HLA-DRB1*04:05  HLA-DRB4*01:01  HLA-DRB1*07:01  HLA-DRB1*04:01  HLA-DRB1*09:01 |
|  | KRSFIEDLLFNKVTL | HLA-DPA1*01:03/DPB1*02:01  HLA-DPA1*02:01/DPB1*01:01  HLA-DPA1*03:01/DPB1*04:02 |
|  | LQIPFAMQMAYRFNG | HLA-DRB1*01:01  HLA-DRB5*01:01  HLA-DRB1*07:01  HLA-DRB1*15:01  HLA-DRB1*11:01  HLA-DRB1*09:01  HLA-DRB4*01:01  HLA-DRB1*01:01 |
|  | NCTFEYVSQPFLMDL | HLA-DPA1*01:03/DPB1*02:01  HLA-DPA1*02:01/DPB1*01:01  HLA-DRB1*01:01  HLA-DPA1*03:01/DPB1*04:02  HLA-DRB1*07:01 |
|  | NQKLIANQFNSAIGK | HLA-DRB1*13:02  HLA-DRB3*02:02  HLA-DRB1*01:01 |
|  | NSVAYSNNSIAIPTN | HLA-DRB1*13:02  HLA-DRB3*02:02 |
|  | NYNYLYRLFRKSNLK | HLA-DRB5*01:01  HLA-DRB1*15:01  HLA-DRB1*01:01 |
|  | PIGINITRFQTLLAL | HLA-DRB1*15:01  HLA-DRB1*01:01  HLA-DPA1*01:03/DPB1*02:01  HLA-DRB1*13:02  HLA-DRB4*01:01 |
|  | PINLVRDLPQGFSAL | HLA-DRB1*03:01  HLA-DRB3*01:01  HLA-DRB1*13:02 |
|  | PTNFTISVTTEILPV | HLA-DRB1*07:01  HLA-DRB1*01:01  HLA-DRB1*09:01 |
|  | QMAYRFNGIGVTQNV | HLA-DRB1*01:01  HLA-DRB3*02:02 |
|  | QPYRVVVLSFELLHA | HLA-DPA1*01:03/DPB1*02:01  HLA-DPA1*02:01/DPB1*01:01  HLA-DPA1*03:01/DPB1*04:02 |
|  | QQLIRAAEIRASANL | \| HLA-DRB1*01:01 \|  \| \| --- \| --- \| \| HLA-DRB4*01:01 \|  \| \| HLA-DQA1*05:01/DQB1*03:01 \| \| \| HLA-DQA1*01:02/DQB1*06:02 \| \| \| HLA-DRB1*08:02 \|  \| \| HLA-DRB5*01:01 \|  \| |
|  | QSLLIVNNATNVVIK | \| HLA-DRB1*13:02 \| \| --- \| \| HLA-DRB3*02:02 \| \| HLA-DRB1*01:01 \| \| HLA-DRB1*04:01 \| \| HLA-DRB1*07:01 \| |
|  | QTYVTQQLIRAAEIR | HLA-DRB1*01:01  HLA-DRB4*01:01 |
|  | REGVFVSNGTHWFVT | HLA-DRB1*13:02  HLA-DRB3*02:02  HLA-DRB1*07:01  HLA-DRB1*01:01  HLA-DRB1*09:01 |
|  | RGVYYPDKVFRSSVL | \| HLA-DRB3*01:01 \| \| --- \| \| HLA-DRB1*01:01 \| |
|  | SEFRVYSSANNCTFE | HLA-DRB1*01:01  HLA-DRB1*09:01 |
|  | SFVIRGDEVRQIAPG | HLA-DRB1*03:01  HLA-DRB3*01:01 |
|  | SSGWTAGAAAYYVGY | HLA-DQA1*05:01/DQB1*03:01  HLA-DRB1*09:01  HLA-DRB1*01:01  HLA-DQA1*01:02/DQB1*06:02 |
|  | TGRLQSLQTYVTQQL | HLA-DRB1*01:01  HLA-DRB1*15:01 |
|  | TLLALHRSYLTPGDS | HLA-DRB1*01:01  HLA-DRB1*15:01 |
|  | TSNFRVQPTESIVRF | HLA-DRB1*01:01  HLA-DRB1*04:01  HLA-DRB1*07:01  HLA-DRB1*04:05  HLA-DRB1*13:02  HLA-DRB1*09:01 |
|  | TVEKGIYQTSNFRVQ | HLA-DRB1*07:01  HLA-DRB1*13:02  HLA-DRB1*01:01 |
|  | TWRVYSTGSNVFQTR | HLA-DRB1*07:01  HLA-DRB1*01:01  HLA-DRB1*09:01 |
|  | VKQLSSNFGAISSVL | HLA-DRB1*13:02  HLA-DRB1*01:01  HLA-DRB3*02:02  HLA-DQA1*05:01/DQB1*03:01 |
|  | VLSFELLHAPATVCG | HLA-DRB1*01:01  HLA-DRB1*09:01 |
|  | VNFNFNGLTGTGVLT | HLA-DRB1*01:01  HLA-DRB1*09:01  HLA-DRB1*07:01 |
|  | YFKIYSKHTPINLVR | HLA-DRB1*01:01  HLA-DRB1*07:01  HLA-DRB5*01:01  HLA-DRB1*11:01  HLA-DRB1*09:01 |
|  | YRLFRKSNLKPFERD | HLA-DRB5*01:01  HLA-DRB1*11:01 |
|  | YSVLYNSASFSTFKC | HLA-DRB1*01:01  HLA-DRB1*13:02  HLA-DRB3*02:02  HLA-DRB1*07:01 |
|  | YTSALLAGTITSGWT | HLA-DQA1*05:01/DQB1*03:01  HLA-DRB1*01:01 |
|  | YYVGYLQPRTFLLKY | HLA-DRB1*01:01  HLA-DRB5*01:01  HLA-DRB1*07:01  HLA-DRB1*15:01  HLA-DRB1*09:01  HLA-DPA1*01:03/DPB1*02:01 |

| Membrane protein | AAVYRINWITGGIAI | HLA-DRB1*01:01  HLA-DRB3*02:02  HLA-DRB1*13:02  HLA-DRB1*07:01 |
| --- | --- | --- |
|  | ASFRLFARTRSMWSF | HLA-DRB1*11:01  HLA-DRB5*01:01  HLA-DRB1*01:01  HLA-DRB1*07:01  HLA-DRB1*09:01  HLA-DRB1*04:01  HLA-DRB1*15:01  HLA-DRB1*08:02  HLA-DRB1*04:05  HLA-DRB4*01:01 |
|  | ETNILLNVPLHGTIL | HLA-DRB1*13:02  HLA-DRB1*01:01  HLA-DRB3*02:02  HLA-DRB4*01:01 |
|  | ILRGHLRIAGHHLGR | HLA-DRB1*01:01  HLA-DRB5*01:01  HLA-DRB1*11:01  HLA-DRB4*01:01 |
|  | LLQFAYANRNRFLYI | HLA-DRB1*01:01  HLA-DRB5*01:01  HLA-DRB1*13:02  HLA-DRB1*11:01  HLA-DRB3*02:02  HLA-DRB1*15:01  HLA-DRB1*07:01  HLA-DRB1*09:01 |
|  | LSYFIASFRLFARTR | HLA-DRB1*11:01  HLA-DRB1*01:01  HLA-DRB5*01:01  HLA-DRB1*15:01  HLA-DPA1*01:03/DPB1*02:01  HLA-DRB1*07:01  HLA-DPA1*02:01/DPB1*01:01  HLA-DPA1*03:01/DPB1*04:02  HLA-DRB1*09:01 |
|  | LVIGAVILRGHLRIA | HLA-DRB5*01:01  HLA-DRB1*01:01  HLA-DRB1*11:01  HLA-DRB4*01:01  HLA-DRB1*15:01 |
|  | NWITGGIAIAMACLV | HLA-DQA1*05:01/DQB1*03:01  HLA-DRB1*01:01  HLA-DQA1*01:02/DQB1*06:02 |
|  | PKEITVATSRTLSYY | HLA-DRB1*07:01  HLA-DRB1*01:01  HLA-DRB1*09:01  HLA-DRB1*13:02 |
|  | PVTLACFVLAAVYRI | HLA-DRB1*01:01  HLA-DRB1*07:01 |
|  | TLSYYKLGASQRVAG | HLA-DRB1*01:01  HLA-DRB5*01:01  HLA-DRB1*09:01  HLA-DRB1*07:01  HLA-DRB1*04:01  HLA-DRB1*11:01 |
|  | VGLMWLSYFIASFRL | \| HLA-DPA1*01:03/DPB1*02:01 \| \| --- \| \| HLA-DRB1*15:01 \| \| HLA-DRB1*01:01 \| \| HLA-DRB1*07:01 \| |

| Envelope protein | LVTLAILTALRLCAY | HLA-DRB1*01:01  HLA-DRB1*15:01  HLA-DRB5*01:01  HLA-DRB4*01:01  HLA-DRB1*11:01 |
| --- | --- | --- |
|  | FYVYSRVKNLNSSRV | HLA-DRB1*01:01  HLA-DRB1*11:01  HLA-DRB1*04:01  HLA-DRB1*07:01  HLA-DRB5*01:01  HLA-DRB1*04:05  HLA-DRB1*13:02 |
|  | LVKPSFYVYSRVKNL | HLA-DRB1*07:01  HLA-DRB1*11:01  HLA-DRB5*01:01  HLA-DRB1*01:01 |
|  | RVKNLNSSRVPDLLV | HLA-DRB1*01:01 |
|  | NIVNVSLVKPSFYVY | HLA-DRB1*01:01  HLA-DRB1*07:01 |

| Nucleocapsid protein | ASAFFGMSRIGMEVT | HLA-DRB1*01:01  HLA-DRB1*11:01 |
| --- | --- | --- |
|  | ATKAYNVTQAFGRRG | HLA-DRB5*01:01  HLA-DRB1*01:01  HLA-DRB1*07:01  HLA-DRB1*09:01 |
|  | DQIGYYRRATRRIRG | HLA-DRB5*01:01  HLA-DRB1*11:01  HLA-DRB1*01:01 |
|  | KDQVILLNKHIDAYK | HLA-DRB1*13:02  HLA-DRB1*11:01  HLA-DRB1*01:01  HLA-DRB4*01:01 |
|  | LALLLLDRLNQLESK | HLA-DRB1*03:01  HLA-DRB4*01:01  HLA-DRB1*13:02  HLA-DRB3*01:01 |
|  | NAAIVLQLPQGTTLP | HLA-DRB1*01:01  HLA-DRB4*01:01 |
|  | NTASWFTALTQHGKE | HLA-DRB1*01:01  HLA-DRB5*01:01 |
|  | QIAQFAPSASAFFGM | HLA-DRB1*01:01  HLA-DRB1*09:01  HLA-DRB1*07:01  HLA-DQA1*05:01/DQB1*03:01 |
|  | RWYFYYLGTGPEAGL | HLA-DRB1*01:01  HLA-DRB1*09:01  HLA-DRB1*04:05 |
|  | TPSGTWLTYTGAIKL | HLA-DRB1*07:01  HLA-DRB1*01:01  HLA-DRB1*09:01  HLA-DRB5*01:01 |
|  | WLTYTGAIKLDDKDP | HLA-DRB1*01:01  HLA-DRB1*09:01 |

| **Supp. Table 4b: MHC class I epitopes and their recognizing alleles used for the population coverage analysis.** | | |
| --- | --- | --- |
| Spike protein | AEIRASANL | HLA-B*40:01  HLA-B*44:03  HLA-B*44:02 |
|  | AEVQIDRLI | HLA-B*44:02  HLA-B*44:03 |
|  | ASANLAATK | HLA-A*11:01  HLA-A*30:01  HLA-A*03:01 |
|  | ASFSTFKCY | HLA-A*30:02  HLA-B*15:01 |
|  | CVADYSVLY | HLA-B*35:01  HLA-A*26:01  HLA-A*68:01  HLA-A*01:01  HLA-A*30:02 |
|  | EILPVSMTK | HLA-A*68:01  HLA-A*11:01 |
|  | FAMQMAYRF | HLA-B*35:01  HLA-B*53:01  HLA-B*58:01  HLA-A*23:01 |
|  | FAQVKQIYK | HLA-A*68:01  HLA-A*11:01 |
|  | FASVYAWNR | HLA-A*68:01  HLA-A*33:01  HLA-A*31:01 |
|  | FIAGLIAIV | HLA-A*02:03  HLA-A*02:06  HLA-A*02:01  HLA-A*68:02 |
|  | FLHVTYVPA | HLA-A*02:03  HLA-A*02:01  HLA-A*02:06 |
|  | FNATRFASV | HLA-B*08:01  HLA-A*68:02 |
|  | FQFCNDPFL | HLA-A*02:06  HLA-A*02:01  HLA-A*02:03 |
|  | FSTFKCYGV | HLA-A*68:02  HLA-A*02:06 |
|  | FTISVTTEI | HLA-A*68:02  HLA-A*02:06  HLA-A*02:03  HLA-A*02:01 |
|  | FVFKNIDGY | HLA-B*35:01  HLA-A*26:01 |
|  | FVFLVLLPL | HLA-A*02:06  HLA-A*02:01 |
|  | FVSGNCDVV | HLA-A*02:03  HLA-A*02:06  HLA-A*68:02 |
|  | GSFCTQLNR | HLA-A*11:01  HLA-A*31:01  HLA-A*68:01 |
|  | GTHWFVTQR | HLA-A*31:01  HLA-A*68:01  HLA-A*11:01 |
|  | GTITSGWTF | HLA-B*58:01  HLA-A*32:01 |
|  | GVYFASTEK | HLA-A*11:01  HLA-A*03:01  HLA-A*68:01  HLA-A*30:01 |
|  | GVYYHKNNK | HLA-A*03:01  HLA-A*11:01  HLA-A*30:01 |
|  | HADQLTPTW | HLA-B*58:01  HLA-B*53:01 |
|  | HLMSFPQSA | HLA-A*02:03  HLA-A*02:01 |
|  | IAIPTNFTI | HLA-B*58:01  HLA-A*02:06 |
|  | IPFAMQMAY | HLA-B*35:01  HLA-B*53:01 |
|  | IYQTSNFRV | HLA-A*24:02  HLA-A*23:01 |
|  | KIADYNYKL | HLA-A*02:01  HLA-A*02:06  HLA-A*32:01  HLA-A*02:03 |
|  | KIYSKHTPI | HLA-A*30:01  HLA-A*32:01  HLA-A*02:03  HLA-A*02:06 |
|  | KQIYKTPPI | HLA-A*32:01  HLA-A*02:03  HLA-A*02:01  HLA-A*30:01 |
|  | LAGTITSGW | HLA-B*58:01  HLA-B*57:01  HLA-B*53:01 |
|  | LLALHRSYL | HLA-A*02:03  HLA-B*08:01 |
|  | LLFNKVTLA | HLA-A*02:03  HLA-A*02:01  HLA-A*02:06 |
|  | LPFFSNVTW | HLA-B*53:01  HLA-B*35:01 |
|  | LPFNDGVYF | HLA-B*35:01  HLA-B*53:01 |
|  | LQIPFAMQM | HLA-B*15:01  HLA-A*02:06 |
|  | MIAQYTSAL | HLA-A*02:03  HLA-A*68:02  HLA-B*35:01  HLA-A*02:06  HLA-B*07:02  HLA-B*15:01  HLA-B*08:01  HLA-A*02:01 |
|  | MTSCCSCLK | HLA-A*68:01  HLA-A*11:01  HLA-A*03:01 |
|  | NSASFSTFK | HLA-A*68:01  HLA-A*11:01 |
|  | NTQEVFAQV | HLA-A*68:02  HLA-A*02:06 |
|  | NYLYRLFRK | HLA-A*33:01  HLA-A*31:01  HLA-A*24:02  HLA-A*23:01 |
|  | QIYKTPPIK | HLA-A*03:01  HLA-A*11:01 |
|  | QTNSPRRAR | HLA-A*31:01  HLA-A*68:01 |
|  | QYIKWPWYI | HLA-A*23:01  HLA-A*24:02 |
|  | RLFRKSNLK | HLA-A*03:01  HLA-A*30:01  HLA-A*11:01  HLA-A*31:01 |
|  | RLQSLQTYV | HLA-A*02:03  HLA-A*02:01  HLA-A*02:06 |
|  | RSFIEDLLF | HLA-B*58:01  HLA-B*57:01  HLA-A*32:01 |
|  | RVVVLSFEL | HLA-A*32:01  HLA-A*02:06 |
|  | RVYSTGSNV | HLA-A*30:01  HLA-A*02:03 |
|  | SANNCTFEY | HLA-B*35:01  HLA-A*30:02 |
|  | SIIAYTMSL | HLA-A*02:06  HLA-A*02:03  HLA-A*02:01  HLA-A*32:01  HLA-A*68:02 |
|  | STQDLFLPF | HLA-B*15:01  HLA-A*32:01 |
|  | SVLNDILSR | HLA-A*11:01  HLA-A*68:01 |
|  | SVLYNSASF | HLA-B*15:01  HLA-A*32:01 |
|  | SVTTEILPV | HLA-A*02:06  HLA-A*02:03  HLA-A*68:02 |
|  | SVYAWNRKR | HLA-A*31:01  HLA-A*68:01  HLA-A*33:01 |
|  | TLADAGFIK | HLA-A*11:01  HLA-A*68:01 |
|  | TLKSFTVEK | HLA-A*11:01  HLA-A*03:01  HLA-A*30:01  HLA-A*68:01 |
|  | TSNQVAVLY | HLA-A*01:01  HLA-A*30:02 |
|  | VASQSIIAY | HLA-B*35:01  HLA-B*15:01 |
|  | VLNDILSRL | HLA-A*02:03  HLA-A*02:01  HLA-A*02:06 |
|  | VTWFHAIHV | HLA-A*02:06  HLA-A*68:02 |
|  | VTYVPAQEK | HLA-A*02:06  HLA-A*68:02 |
|  | VVFLHVTYV | HLA-A*02:03  HLA-A*02:06  HLA-A*02:01  HLA-A*68:02 |
|  | VYYPDKVFR | HLA-A*31:01  HLA-A*33:01 |
|  | WTAGAAAYY | HLA-A*26:01  HLA-A*68:01  HLA-A*01:01  HLA-A*30:02  HLA-B*35:01  HLA-B*15:01 |
|  | WTFGAGAAL | HLA-A*68:02  HLA-A*02:06 |
|  | YEQYIKWPW | HLA-B*44:02  HLA-B*44:03 |
|  | YFPLQSYGF | HLA-A*24:02  HLA-A*23:01 |
|  | YLQPRTFLL | HLA-A*02:01  HLA-A*02:03  HLA-A*02:06  HLA-B*08:01 |
|  | YNYLYRLFR | HLA-A*31:01  HLA-A*33:01  HLA-A*68:01 |
|  | YQDVNCTEV | HLA-A*02:06  HLA-A*02:01 |
|  | YSSANNCTF | HLA-B*58:01  HLA-B*35:01  HLA-B*15:01 |
|  | YTNSFTRGV | HLA-A*68:02  HLA-A*02:03  HLA-A*02:06 |
|  | YYVGYLQPR | HLA-A*33:01  HLA-A*31:01 |

| Membrane protein | AMACLVGLM | HLA-A*02:03  HLA-B*15:01 |
| --- | --- | --- |
|  | ATSRTLSYY | HLA-A*30:02  HLA-A*11:01  HLA-A*01:01 |
|  | AVILRGHLR | HLA-A*31:01  HLA-A*68:01 |
|  | CFVLAAVYR | HHLA-A*33:01  HLA-A*31:01 |
|  | DSGFAAYSR | HLA-A*68:01  HLA-A*33:01 |
|  | FIASFRLFA | HLA-A*02:03  HLA-A*68:02  HLA-A*02:06  HLA-A*02:01 |
|  | FLWLLWPVT | HLA-A*02:01  HLA-A*02:06 |
|  | FVLAAVYRI | HLA-A*02:06  HLA-A*02:01  HLA-A*68:02  HLA-A*02:03 |
|  | GLMWLSYFI | HLA-A*02:01  HLA-A*02:06  HLA-A*02:03 |
|  | GTITVEELK | HLA-A*11:01  HLA-A*68:01 |
|  | IASFRLFAR | HLA-A*31:01  HLA-A*68:01  HLA-A*33:01 |
|  | KLIFLWLLW | HLA-A*32:01  HLA-B*58:01 |
|  | KLLEQWNLV | HLA-A*02:01  HLA-A*02:06  HLA-A*02:03 |
|  | LAAVYRINW | HLA-B*58:01  HLA-B*57:01  HLA-B*53:01 |
|  | LACFVLAAV | HLA-A*68:02  HLA-A*02:06 |
|  | LLQFAYANR | HLA-A*31:01  HLA-A*33:01 |
|  | LSYFIASFR | HLA-A*68:01  HLA-A*31:01  HLA-A*33:01  HLA-A*11:01  HLA-A*03:01 |
|  | LWPVTLACF | HLA-A*24:02  HLA-A*23:01 |
|  | MACLVGLMW | HLA-B*58:01  HLA-B*57:01  HLA-B*53:01 |
|  | QFAYANRNR | HLA-A*33:01  HLA-A*31:01 |
|  | RIAGHHLGR | HLA-A*31:01  HLA-A*03:01 |
|  | RLFARTRSM | HLA-A*32:01  HLA-B*08:01  HLA-B*15:01  HLA-A*30:01  HLA-A*02:03  HLA-B*07:02 |
|  | SFRLFARTR | HLA-A*31:01  HLA-A*33:01 |
|  | SMWSFNPET | HLA-A*02:01  HLA-A*02:06 |
|  | SYFIASFRL | HLA-A*23:01  HLA-A*24:02 |
|  | TLACFVLAA | HLA-A*02:03  HLA-A*02:01  HLA-A*02:06 |
|  | TSRTLSYYK | HLA-A*30:01  HLA-A*11:01  HLA-A*31:01  HLA-A*68:01 |
|  | WLLWPVTLA | HLA-A*02:03  HLA-A*02:01  HLA-A*02:06 |
|  | YANRNRFLY | HLA-B*35:01  HLA-A*30:02  HLA-B*58:01 |
|  | YFIASFRLF | HLA-A*23:01  HLA-A*24:02 |

| Nucleocapsid protein | ASAFFGMSR | HLA-A*11:01  HLA-A*68:01  HLA-A*31:01 |
| --- | --- | --- |
|  | FTALTQHGK | HLA-A*68:01  HLA-A*11:01 |
|  | GMSRIGMEV | HLA-A*02:03  HLA-A*02:01 |
|  | IGYYRRATR | HLA-A*31:01  HLA-A*33:01 |
|  | KAYNVTQAF | HLA-A*32:01  HLA-B*58:01  HLA-B*15:01  HLA-B*57:01  HLA-B*35:01 |
|  | KMKDLSPRW | HLA-A*32:01  HLA-B*58:01 |
|  | KTFPPTEPK | HLA-A*11:01  HLA-A*30:01  HLA-A*03:01  HLA-A*31:01  HLA-A*68:01 |
|  | LLLDRLNQL | HLA-A*02:03  HLA-A*02:01  HLA-A*02:06 |
|  | LPAADLDDF | HLA-B*35:01  HLA-B*53:01 |
|  | LPNNTASWF | HLA-B*35:01  HLA-B*53:01 |
|  | LSPRWYFYY | HLA-A*30:02  HLA-A*01:01 |
|  | MEVTPSGTW | HLA-B*44:02  HLA-B*44:03 |
|  | NTASWFTAL | HLA-A*68:02  HLA-A*02:06 |
|  | SPRWYFYYL | HLA-B*07:02  HLA-B*08:01 |
|  | SSRGTSPAR | HLA-A*31:01  HLA-A*30:01 |
|  | SSRSRNSSR | HLA-A*31:01  HLA-A*30:01 |
|  | SSRSSSRSR | HHLA-A*30:01  HLA-A*31:01 |
|  | TPSGTWLTY | HLA-B*35:01  HLA-B*53:01 |

| Envelope protein | FLAFVVFLL | HLA-A*02:01  HLA-A*02:03  HLA-A*02:06  HLA-A*68:02 |
| --- | --- | --- |
|  | FLLVTLAIL | HLA-A*02:01  HLA-A*02:03  HLA-A*02:06 |
|  | FVSEETGTL | HLA-A*02:03 |
|  | IVNSVLLFL | HLA-A*02:06  HLA-A*02:03 |
|  | LIVNSVLLF | HLA-B*15:01 |
|  | LTALRLCAY | HLA-B*15:01 |
|  | LVKPSFYVY | HLA-B*15:01  HLA-A*30:02 |
|  | NIVNVSLVK | HLA-A*68:01  HLA-A*11:01 |
|  | RLCAYCCNI | HLA-A*02:03 |
|  | RVKNLNSSR | HLA-A*31:01  HLA-A*30:01 |
|  | SFYVYSRVK | HLA-A*30:01 |
|  | SLVKPSFYV | HLA-A*02:01  HLA-A*02:03  HLA-A*02:06  HLA-A*68:02 |
|  | SSRVPDLLV | HLA-A*30:01 |
|  | SVLLFLAFV | HLA-A*02:06  HLA-A*02:01  HLA-A*68:02  HLA-A*02:03 |
|  | TLAILTALR | HLA-A*68:01  HLA-A*33:01  HLA-A*31:01 |
|  | VLLFLAFVV | HLA-A*02:01  HLA-A*02:06 |
|  | VSLVKPSFY | HLA-A*30:02 |
|  | VTLAILTAL | HLA-A*02:06 |
|  | YVYSRVKNL | HLA-A*02:03  HLA-B*08:01 |
