# Supplementary material for: Population-Predicted MHC Class II Epitope Presentation of SARS-CoV-2 Structural Proteins Correlates to the Case Fatality Rates of COVID-19 in Different Countries
Source: Int J Mol Sci. 2021 Mar 5;22(5):2630. doi: 10.3390/ijms22052630 (PMC7961590; doi:10.3390/ijms22052630)
Supplement: Supplementary file 1 [file ijms-22-02630-s001.zip › TableS5_Population.docx]

**Supp. Table S5: Population coverage for MHC I and MHC II alleles**

**Population coverage for MHC class I alleles**

| COUNTRY | Nucleocapsid protein | Spike protein | Membrane protein | Envelope protein |
| --- | --- | --- | --- | --- |
| American Samoa | 70.35% | 98.75% | 97.63% | 67.51% |
| American Samoa Polynesian | 70.35% | 98.75% | 97.63% | 67.51% |
| Argentina | 95.81% | 97.24% | 97.01% | 90.57% |
| Argentina Amerindian | 95.81% | 97.24% | 97.01% | 90.57% |
| Australia | 80.33% | 94.27% | 93.16% | 60.02% |
| Australia Australian Aborigines | 59.76% | 87.25% | 86.9% | 49.87% |
| Australia Caucasoid | 99.28% | 99.92% | 99.75% | 81.09% |
| Austria | 98.84% | 99.89% | 99.59% | 80.88% |
| Austria Caucasoid | 98.84% | 99.89% | 99.59% | 80.88% |
| Belgium | 97.84% | 99.39% | 99.21% | 76.3% |
| Belgium Caucasoid | 97.84% | 99.39% | 99.21% | 76.3% |
| Brazil | 89.68% | 95.66% | 94.0% | 70.81% |
| Brazil Amerindian | 88.7% | 93.11% | 93.11% | 88.3% |
| Brazil Caucasoid | 94.38% | 99.11% | 98.15% | 74.94% |
| Brazil Mixed | 91.54% | 96.49% | 95.01% | 69.05% |
| Bulgaria | 92.06% | 99.01% | 97.53% | 69.34% |
| Bulgaria Caucasoid | 91.35% | 99.12% | 97.47% | 70.44% |
| Bulgaria Other | 97.06% | 99.58% | 98.93% | 71.76% |
| Burkina Faso | 65.07% | 67.18% | 66.28% | 35.47% |
| Burkina Faso Black | 65.07% | 67.18% | 66.28% | 35.47% |
| Cameroon | 81.91% | 89.24% | 85.65% | 46.06% |
| Cameroon Black | 81.91% | 89.24% | 85.65% | 46.06% |
| Cape Verde | 94.82% | 98.86% | 98.15% | 70.62% |
| Cape Verde Black | 94.82% | 98.86% | 98.15% | 70.62% |
| Central Africa | 78.18% | 85.22% | 81.8% | 46.58% |
| Central African Republic | 28.02% | 28.02% | 15.72% | 7.99% |
| Central African Republic Black | 28.02% | 28.02% | 15.72% | 7.99% |
| Central America | 7.01% | 7.76% | 7.76% | 2.78% |
| Chile | 85.82% | 95.6% | 93.45% | 66.18% |
| Chile Amerindian | 85.76% | 99.63% | 99.41% | 79.52% |
| Chile Mixed | 80.98% | 89.12% | 85.3% | 56.55% |
| China | 84.75% | 93.99% | 92.83% | 77.2% |
| China Oriental | 84.75% | 93.99% | 92.83% | 77.2% |
| Colombia | 8.36% | 8.36% | 8.36% | 8.36% |
| Colombia Black | 3.65% | 3.65% | 3.65% | 3.65% |
| Colombia Mestizo | 14.07% | 14.07% | 14.07% | 14.07% |
| Croatia | 95.67% | 99.68% | 98.96% | 70.69% |
| Croatia Caucasoid | 95.67% | 99.68% | 98.96% | 70.69% |
| Cuba | 93.85% | 98.66% | 97.48% | 67.68% |
| Cuba Caucasoid | 93.57% | 98.81% | 97.58% | 67.95% |
| Cuba Mulatto | 94.27% | 98.46% | 97.46% | 67.19% |
| Czech Republic | 97.87% | 98.8% | 97.65% | 69.99% |
| Czech Republic Caucasoid | 97.87% | 98.8% | 97.65% | 69.99% |
| East Africa | 84.98% | 90.18% | 88.83% | 62.46% |
| East Asia | 83.13% | 97.65% | 95.22% | 68.95% |
| Ecuador | 22.72% | 77.35% | 76.97% | 22.72% |
| Ecuador Amerindian | 22.72% | 77.35% | 76.97% | 22.72% |
| England | 99.39% | 99.91% | 99.71% | 80.02% |
| England Caucasoid | 99.39% | 99.91% | 99.71% | 80.02% |
| Europe | 97.79% | 99.61% | 99.01% | 75.58% |
| Finland | 99.61% | 99.99% | 99.97% | 84.44% |
| Finland Caucasoid | 99.61% | 99.99% | 99.97% | 84.44% |
| France | 98.01% | 99.74% | 99.17% | 73.15% |
| France Caucasoid | 98.01% | 99.74% | 99.17% | 73.15% |
| Georgia | 90.25% | 97.5% | 95.7% | 67.45% |
| Georgia Caucasoid | 92.38% | 98.32% | 97.13% | 71.01% |
| Georgia Kurd | 85.32% | 97.42% | 91.41% | 54.29% |
| Germany | 99.05% | 99.91% | 99.69% | 78.58% |
| Germany Caucasoid | 99.05% | 99.91% | 99.69% | 78.58% |
| Guatemala | 7.01% | 7.76% | 7.76% | 2.78% |
| Guatemala Amerindian | 7.01% | 7.76% | 7.76% | 2.78% |
| Guinea-Bissau | 89.94% | 96.18% | 94.89% | 57.68% |
| Guinea-Bissau Black | 89.94% | 96.18% | 94.89% | 57.68% |
| Hong Kong | 83.61% | 95.53% | 94.86% | 77.4% |
| Hong Kong Oriental | 83.61% | 95.53% | 94.86% | 77.4% |
| India | 80.79% | 88.22% | 86.31% | 56.51% |
| India Asian | 80.79% | 88.22% | 86.31% | 56.51% |
| Indonesia | 72.83% | 85.66% | 81.31% | 53.25% |
| Indonesia Austronesian | 72.83% | 85.66% | 81.31% | 53.25% |
| Iran | 94.26% | 96.76% | 95.26% | 78.48% |
| Iran Persian | 94.26% | 96.76% | 95.26% | 78.48% |
| Ireland Northern | 99.6% | 99.93% | 99.72% | 79.94% |
| Ireland Northern Caucasoid | 99.6% | 99.93% | 99.72% | 79.94% |
| Ireland South | 99.57% | 99.92% | 99.65% | 77.68% |
| Ireland South Caucasoid | 99.57% | 99.92% | 99.65% | 77.68% |
| Israel | 78.65% | 88.68% | 83.54% | 47.98% |
| Israel Arab | 83.78% | 93.41% | 89.38% | 55.56% |
| Israel Jew | 84.83% | 93.58% | 89.11% | 50.79% |
| Italy | 96.08% | 98.42% | 97.11% | 66.22% |
| Italy Caucasoid | 96.08% | 98.42% | 97.11% | 66.22% |
| Ivory Coast | 65.09% | 65.09% | 60.97% | 4.54% |
| Ivory Coast Black | 65.09% | 65.09% | 60.97% | 4.54% |
| Japan | 82.34% | 98.2% | 95.94% | 69.33% |
| Japan Oriental | 82.34% | 98.2% | 95.94% | 69.33% |
| Jordan | 82.04% | 89.28% | 86.34% | 61.86% |
| Jordan Arab | 82.04% | 89.28% | 86.34% | 61.86% |
| Kenya | 84.08% | 88.75% | 88.01% | 58.34% |
| Kenya Black | 84.08% | 88.75% | 88.01% | 58.34% |
| Korea; South | 88.14% | 97.65% | 95.23% | 74.46% |
| Korea; South Oriental | 88.14% | 97.65% | 95.23% | 74.46% |
| Macedonia | 26.72% | 26.72% | 26.72% | 14.07% |
| Macedonia Caucasoid | 26.72% | 26.72% | 26.72% | 14.07% |
| Malaysia | 69.71% | 79.44% | 78.81% | 61.45% |
| Malaysia Austronesian | 45.3% | 56.97% | 56.97% | 26.04% |
| Malaysia Oriental | 76.83% | 86.23% | 85.74% | 70.21% |
| Mali | 87.92% | 95.67% | 95.46% | 61.36% |
| Mali Black | 87.92% | 95.67% | 95.46% | 61.36% |
| Martinique | N/A | 22.56% | 22.56% | N/A |
| Martinique Black | N/A | 22.56% | 22.56% | N/A |
| Mexico | 91.65% | 97.82% | 97.25% | 84.18% |
| Mexico Amerindian | 96.64% | 99.86% | 99.85% | 93.63% |
| Mexico Mestizo | 91.87% | 97.89% | 97.22% | 79.3% |
| Mongolia | 80.38% | 95.31% | 94.69% | 65.79% |
| Mongolia Oriental | 80.38% | 95.31% | 94.69% | 65.79% |
| Morocco | 95.14% | 98.43% | 97.12% | 74.19% |
| Morocco Arab | 96.83% | 99.2% | 98.37% | 78.62% |
| Morocco Caucasoid | 93.6% | 97.67% | 96.13% | 70.78% |
| New Caledonia | 44.12% | 98.1% | 97.81% | 36.98% |
| New Caledonia Melanesian | 44.12% | 98.1% | 97.81% | 36.98% |
| North Africa | 89.28% | 95.49% | 94.29% | 66.08% |
| North America | 94.53% | 98.9% | 98.11% | 76.74% |
| Northeast Asia | 84.48% | 94.1% | 93.01% | 77.25% |
| Oceania | 70.9% | 94.64% | 93.87% | 56.28% |
| Oman | 96.01% | 99.43% | 97.81% | 77.75% |
| Oman Arab | 96.01% | 99.43% | 97.81% | 77.75% |
| Pakistan | 88.87% | 97.09% | 94.04% | 71.77% |
| Pakistan Asian | 89.17% | 96.75% | 93.45% | 74.38% |
| Pakistan Mixed | 88.29% | 97.73% | 95.12% | 65.91% |
| Papua New Guinea | 59.85% | 97.92% | 97.59% | 59.65% |
| Papua New Guinea Melanesian | 59.85% | 97.92% | 97.59% | 59.65% |
| Peru | 93.83% | 99.99% | 99.98% | 91.7% |
| Peru Amerindian | 93.83% | 99.99% | 99.98% | 91.7% |
| Philippines | 65.19% | 94.98% | 94.98% | 65.19% |
| Philippines Austronesian | 65.19% | 94.98% | 94.98% | 65.19% |
| Poland | 97.87% | 99.73% | 99.15% | 76.79% |
| Poland Caucasoid | 97.87% | 99.73% | 99.15% | 76.79% |
| Portugal | 93.56% | 98.28% | 96.78% | 70.67% |
| Portugal Caucasoid | 93.56% | 98.28% | 96.78% | 70.67% |
| Romania | 96.11% | 99.53% | 98.91% | 72.03% |
| Romania Caucasoid | 96.11% | 99.53% | 98.91% | 72.03% |
| Russia | 91.47% | 99.16% | 98.16% | 66.59% |
| Russia Other | 94.18% | 99.98% | 99.54% | 67.92% |
| Russia Siberian | 92.58% | 99.29% | 98.47% | 68.78% |
| Rwanda | 13.11% | 24.87% | 24.87% | 2.97% |
| Rwanda Black | 13.11% | 24.87% | 24.87% | 2.97% |
| Sao Tome and Principe | 92.6% | 96.15% | 95.57% | 66.64% |
| Sao Tome and Principe Black | 92.6% | 96.15% | 95.57% | 66.64% |
| Saudi Arabia | 92.15% | 97.4% | 96.43% | 71.45% |
| Saudi Arabia Arab | 92.15% | 97.4% | 96.43% | 71.45% |
| Scotland | 61.91% | 65.34% | 42.59% | 30.06% |
| Scotland Caucasoid | 61.91% | 65.34% | 42.59% | 30.06% |
| Senegal | 86.98% | 95.35% | 93.99% | 63.52% |
| Senegal Black | 86.98% | 95.35% | 93.99% | 63.52% |
| Serbia | 57.23% | 59.17% | 48.59% | 19.36% |
| Serbia Caucasoid | 57.23% | 59.17% | 48.59% | 19.36% |
| Singapore | 78.66% | 91.93% | 90.6% | 68.47% |
| Singapore Austronesian | 75.62% | 90.16% | 87.67% | 56.87% |
| Singapore Oriental | 82.28% | 94.04% | 93.25% | 75.28% |
| South Africa | 85.34% | 92.53% | 90.47% | 62.45% |
| South Africa Black | 86.71% | 91.96% | 88.75% | 62.57% |
| South Africa Other | 90.44% | 96.88% | 95.81% | 64.65% |
| South America | 75.54% | 87.73% | 85.94% | 61.51% |
| South Asia | 87.37% | 94.14% | 91.76% | 65.59% |
| Southeast Asia | 74.39% | 94.15% | 92.73% | 65.04% |
| Southwest Asia | 83.96% | 91.07% | 87.86% | 61.05% |
| Spain | 80.01% | 84.12% | 79.68% | 62.28% |
| Spain Caucasoid | 80.01% | 84.12% | 79.68% | 62.28% |
| Sri Lanka | 52.39% | 52.39% | 52.39% | 36.16% |
| Sri Lanka Asian | 52.39% | 52.39% | 52.39% | 36.16% |
| Sudan | 86.64% | 92.93% | 91.83% | 65.04% |
| Sudan Arab | 57.07% | 70.21% | 66.99% | 42.78% |
| Sudan Black | 2.19% | 2.19% | 2.19% | N/A |
| Sudan Mixed | 87.8% | 93.5% | 92.46% | 66.14% |
| Sweden | 98.74% | 99.99% | 99.92% | 75.77% |
| Sweden Caucasoid | 98.74% | 99.99% | 99.92% | 75.77% |
| Thailand | 85.78% | 90.25% | 87.96% | 74.28% |
| Thailand Oriental | 85.78% | 90.25% | 87.96% | 74.28% |
| Tunisia | 90.9% | 97.01% | 96.75% | 68.92% |
| Tunisia Arab | 90.9% | 97.01% | 96.75% | 68.92% |
| Turkey | 44.8% | 44.8% | 44.8% | 44.8% |
| Turkey Caucasoid | 44.8% | 44.8% | 44.8% | 44.8% |
| Uganda | 90.09% | 94.43% | 93.07% | 68.37% |
| Uganda Black | 90.09% | 94.43% | 93.07% | 68.37% |
| United Arab Emirates | 2.19% | 3.37% | 3.37% | 2.19% |
| United Arab Emirates Arab | 2.19% | 3.37% | 3.37% | 2.19% |
| United States | 94.72% | 98.95% | 98.17% | 76.78% |
| United States Amerindian | 90.07% | 99.62% | 99.5% | 80.91% |
| United States Asian | 86.68% | 96.9% | 95.32% | 71.15% |
| United States Black | 93.0% | 96.9% | 95.67% | 67.13% |
| United States Caucasoid | 98.57% | 99.78% | 99.38% | 77.4% |
| United States Hispanic | 94.02% | 98.72% | 97.75% | 76.1% |
| United States Mestizo | 94.67% | 99.09% | 98.49% | 77.68% |
| United States Polynesian | 84.18% | 99.42% | 97.49% | 73.15% |
| Venezuela | 66.97% | 89.91% | 89.91% | 65.02% |
| Venezuela Amerindian | 68.52% | 89.94% | 89.94% | 67.34% |
| Venezuela Caucasoid | 2.39% | 11.45% | 11.45% | N/A |
| Venezuela Mestizo | 1.99% | 9.75% | 9.75% | N/A |
| Vietnam | 79.33% | 91.01% | 89.01% | 68.02% |
| Vietnam Oriental | 79.33% | 91.01% | 89.01% | 68.02% |
| Wales | 1.0% | 1.0% | 1.0% | N/A |
| Wales Caucasoid | 1.0% | 1.0% | 1.0% | N/A |
| West Africa | 87.29% | 95.06% | 93.81% | 60.97% |
| West Indies | 93.62% | 98.73% | 97.6% | 67.12% |
| Zambia | 95.03% | 97.76% | 97.33% | 86.14% |
| Zambia Black | 95.03% | 97.76% | 97.33% | 86.14% |
| Zimbabwe | 87.48% | 93.72% | 91.68% | 70.55% |
| Zimbabwe Black | 87.48% | 93.72% | 91.68% | 70.55% |

**Population coverage for MHC class II alleles**

| COUNTRY | Nucleocapsid protein | Spike protein | Membrane protein | Envelope protein |
| --- | --- | --- | --- | --- |
| Algeria | 66,24% | 75,83% | 61,65% | 60,20% |
| Algeria Arab | 66,24% | 75,83% | 61,65% | 60,20% |
| Argentina | 42,63% | 61,58% | 53,84% | 39,10% |
| Argentina Amerindian | 18,69% | 44,48% | 41,93% | 14,80% |
| Argentina Caucasoid | 66,83% | 79,76% | 68,76% | 63,41% |
| Australia | 17,83% | 24,23% | 18,48% | 18,48% |
| Australia Australian Aborigines | 17,83% | 24,23% | 18,48% | 18,48% |
| Austria | 78,19% | 92,71% | 84,95% | 84,16% |
| Austria Caucasoid | 78,19% | 92,71% | 84,95% | 84,16% |
| Belarus | 27,09% | 43,81% | 43,81% | 43,81% |
| Belarus Caucasoid | 27,09% | 43,81% | 43,81% | 43,81% |
| Belgium | 72,44% | 78,47% | 61,44% | 61,44% |
| Belgium Caucasoid | 72,44% | 78,47% | 61,44% | 61,44% |
| Bolivia | 40,71% | 77,82% | 76,38% | 3,96% |
| Bolivia Amerindian | 40,71% | 77,82% | 76,38% | 3,96% |
| Borneo | 13,88% | 42,09% | 38,38% | 38,38% |
| Borneo Austronesian | 13,88% | 42,09% | 38,38% | 38,38% |
| Brazil | 40,01% | 62,86% | 55,47% | 29,77% |
| Brazil Amerindian | 11,17% | 48,43% | 47,90% | 4,32% |
| Brazil Caucasoid | 74,81% | 83,63% | 75,29% | 63,75% |
| Brazil Mixed | 67,90% | 76,14% | 60,28% | 56,30% |
| Brazil Mulatto | 66,24% | 70,30% | 60,44% | 54,17% |
| Bulgaria | 53,62% | 57,23% | 45,83% | 45,83% |
| Bulgaria Caucasoid | 53,62% | 57,23% | 45,83% | 45,83% |
| Cameroon | 41,17% | 41,17% | 29,78% | 29,10% |
| Cameroon Black | 41,17% | 41,17% | 29,78% | 29,10% |
| Canada | 10,94% | 27,22% | 23,33% | 13,10% |
| Canada Amerindian | 10,94% | 27,22% | 23,33% | 13,10% |
| Cape Verde | 73,06% | 78,93% | 70,89% | 64,00% |
| Cape Verde Black | 73,06% | 78,93% | 70,89% | 64,00% |
| Central Africa | 55,26% | 58,06% | 49,06% | 47,36% |
| Central African Republic | 68,75% | 68,75% | 53,21% | 53,21% |
| Central African Republic Black | 68,75% | 68,75% | 53,21% | 53,21% |
| Central America | 24,28% | 49,36% | 46,71% | 23,76% |
| Chile | 43,18% | 64,88% | 58,54% | 43,94% |
| Chile Amerindian | 61,68% | 67,40% | 67,40% | 34,55% |
| Chile Mixed | 34,18% | 51,27% | 43,84% | 35,94% |
| China | 43,85% | 56,04% | 51,69% | 38,46% |
| China Oriental | 43,85% | 56,04% | 51,69% | 38,46% |
| Colombia | 31,37% | 52,37% | 46,68% | 28,81% |
| Colombia Amerindian | 22,07% | 46,96% | 42,05% | 19,38% |
| Colombia Black | 46,27% | 59,83% | 50,35% | 41,86% |
| Colombia Mestizo | 38,38% | 53,08% | 45,39% | 44,20% |
| Congo | 62,20% | 63,54% | 56,27% | 53,17% |
| Congo Black | 62,20% | 63,54% | 56,27% | 53,17% |
| Costa Rica | 19,00% | 24,31% | 22,56% | 17,19% |
| Costa Rica Mestizo | 19,00% | 24,31% | 22,56% | 17,19% |
| Croatia | 60,56% | 64,44% | 52,95% | 52,95% |
| Croatia Caucasoid | 60,56% | 64,44% | 52,95% | 52,95% |
| Cuba | 75,50% | 85,48% | 79,75% | 75,50% |
| Cuba Mixed | 75,50% | 85,48% | 79,75% | 75,50% |
| Czech Republic | 69,06% | 83,07% | 75,69% | 75,27% |
| Czech Republic Caucasoid | 70,80% | 85,42% | 77,76% | 77,29% |
| Czech Republic Other | 56,65% | 64,14% | 60,47% | 60,47% |
| Denmark | 50,58% | 87,68% | 79,48% | 79,48% |
| Denmark Caucasoid | 50,58% | 87,68% | 79,48% | 79,48% |
| East Africa | 61,93% | 62,18% | 53,08% | 51,00% |
| East Asia | 66,84% | 78,57% | 76,32% | 60,02% |
| Ecuador | 36,73% | 52,17% | 52,17% | N/A |
| Ecuador Amerindian | 36,73% | 52,17% | 52,17% | N/A |
| England | 71,06% | 92,88% | 83,79% | 82,50% |
| England Caucasoid | 71,06% | 92,88% | 83,79% | 82,50% |
| Equatorial Guinea | 43,90% | 43,90% | 34,07% | 33,09% |
| Equatorial Guinea Black | 43,90% | 43,90% | 34,07% | 33,09% |
| Ethiopia | 79,44% | 82,77% | 74,42% | 74,42% |
| Ethiopia Black | 79,44% | 82,77% | 74,42% | 74,42% |
| Europe | 67,91% | 84,13% | 73,82% | 72,34% |
| Finland | 28,94% | 51,14% | 42,24% | 42,24% |
| Finland Caucasoid | 28,94% | 51,14% | 42,24% | 42,24% |
| France | 68,26% | 87,63% | 77,01% | 76,71% |
| France Caucasoid | 68,26% | 87,63% | 77,01% | 76,71% |
| Gabon | 41,78% | 41,78% | 41,78% | 37,59% |
| Gabon Black | 41,78% | 41,78% | 41,78% | 37,59% |
| Georgia | 64,81% | 74,45% | 64,81% | 63,25% |
| Georgia Caucasoid | 64,81% | 74,45% | 64,81% | 63,25% |
| Germany | 70,35% | 89,93% | 81,87% | 80,98% |
| Germany Caucasoid | 70,35% | 89,93% | 81,87% | 80,98% |
| Greece | 58,72% | 65,84% | 56,49% | 55,35% |
| Greece Caucasoid | 58,72% | 65,84% | 56,49% | 55,35% |
| Guatemala | 13,70% | 48,59% | 45,24% | 12,39% |
| Guatemala Amerindian | 13,70% | 48,59% | 45,24% | 12,39% |
| Guinea-Bissau | 69,42% | 70,30% | 65,07% | 55,38% |
| Guinea-Bissau Black | 69,42% | 70,30% | 65,07% | 55,38% |
| India | 61,72% | 73,30% | 63,87% | 62,12% |
| India Asian | 61,72% | 73,30% | 63,87% | 62,12% |
| Indonesia | 38,72% | 45,66% | 41,49% | 38,16% |
| Indonesia Austronesian | 38,72% | 45,66% | 41,49% | 38,16% |
| Iran | 57,49% | 63,98% | 51,28% | 49,94% |
| Iran Kurd | 47,44% | 55,11% | 43,75% | 43,75% |
| Iran Persian | 59,55% | 65,72% | 53,01% | 51,57% |
| Ireland Northern | 71,24% | 94,03% | 83,74% | 83,10% |
| Ireland Northern Caucasoid | 71,24% | 94,03% | 83,74% | 83,10% |
| Ireland South | 69,53% | 92,50% | 80,30% | 80,30% |
| Ireland South Caucasoid | 69,53% | 92,50% | 80,30% | 80,30% |
| Israel | 63,09% | 67,14% | 57,41% | 56,46% |
| Israel Arab | 60,91% | 66,70% | 56,83% | 54,68% |
| Israel Jew | 64,15% | 67,69% | 57,99% | 57,61% |
| Italy | 83,10% | 84,66% | 42,36% | 42,09% |
| Italy Caucasoid | 83,10% | 84,66% | 42,36% | 42,09% |
| Jamaica | 17,37% | 27,41% | 23,44% | 23,44% |
| Jamaica Black | 17,37% | 27,41% | 23,44% | 23,44% |
| Japan | 59,43% | 71,62% | 71,43% | 52,24% |
| Japan Oriental | 59,43% | 71,62% | 71,43% | 52,24% |
| Jordan | 21,27% | 52,22% | 49,92% | 49,63% |
| Jordan Arab | 21,27% | 52,22% | 49,92% | 49,63% |
| Kiribati | 3,17% | 9,37% | 9,37% | 9,37% |
| Kiribati Micronesian | 3,17% | 9,37% | 9,37% | 9,37% |
| Korea; South | 71,54% | 81,85% | 79,68% | 67,46% |
| Korea; South Oriental | 71,54% | 81,85% | 79,68% | 67,46% |
| Lebanon | 59,11% | 69,40% | 61,98% | 61,09% |
| Lebanon Arab | 59,11% | 69,40% | 61,98% | 61,09% |
| Macedonia | 54,53% | 65,47% | 56,14% | 55,57% |
| Macedonia Caucasoid | 54,53% | 65,47% | 56,14% | 55,57% |
| Malaysia | 45,89% | 56,35% | 50,23% | 39,13% |
| Malaysia Austronesian | 43,34% | 53,51% | 46,62% | 40,40% |
| Malaysia Oriental | 58,57% | 70,35% | 65,22% | 40,19% |
| Martinique | 63,77% | 70,44% | 63,77% | 60,18% |
| Martinique Black | 63,77% | 70,44% | 63,77% | 60,18% |
| Mexico | 27,83% | 54,39% | 48,87% | 22,52% |
| Mexico Amerindian | 13,84% | 42,59% | 41,55% | 9,85% |
| Mexico Mestizo | 42,02% | 67,79% | 59,47% | 40,69% |
| Mongolia | 63,75% | 78,18% | 68,28% | 59,48% |
| Mongolia Oriental | 63,75% | 78,18% | 68,28% | 59,48% |
| Morocco | 74,31% | 83,03% | 67,26% | 65,91% |
| Morocco Arab | 77,89% | 84,55% | 67,77% | 66,03% |
| Morocco Caucasoid | 65,43% | 79,75% | 67,85% | 67,28% |
| Netherlands | 67,27% | 82,53% | 71,46% | 70,28% |
| Netherlands Caucasoid | 67,27% | 82,53% | 71,46% | 70,28% |
| New Caledonia | 63,90% | 81,41% | 81,41% | 79,38% |
| New Caledonia Melanesian | 63,90% | 81,41% | 81,41% | 79,38% |
| Niue | 30,28% | 40,56% | 59,73% | 19,00% |
| Niue Polynesian | 30,28% | 40,56% | 59,73% | 19,00% |
| North Africa | 65,97% | 74,18% | 60,85% | 59,39% |
| North America | 66,23% | 86,46% | 76,66% | 72,82% |
| Northeast Asia | 43,85% | 56,04% | 51,69% | 38,46% |
| Norway | 68,64% | 93,75% | 84,79% | 84,00% |
| Norway Caucasoid | 68,64% | 93,75% | 84,79% | 84,00% |
| Oceania | 40,06% | 52,94% | 52,09% | 45,07% |
| Pakistan | 1,18% | 1,18% | 1,18% | 1,18% |
| Pakistan Asian | 1,45% | 1,45% | 1,45% | 1,45% |
| Pakistan Mixed | 0,00% | 0,00% | 0,00% | 0,00% |
| Papua New Guinea | 49,31% | 66,39% | 66,39% | 61,33% |
| Papua New Guinea Melanesian | 49,31% | 66,39% | 66,39% | 61,33% |
| Paraguay | 0,00% | 4,90% | 4,90% | 0,00% |
| Paraguay Amerindian | 0,00% | 4,90% | 4,90% | 0,00% |
| Peru | 23,23% | 49,87% | 47,74% | 4,48% |
| Peru Amerindian | 23,23% | 49,87% | 47,74% | 4,48% |
| Philippines | 26,50% | 27,53% | 25,52% | 21,36% |
| Philippines Austronesian | 26,50% | 27,53% | 25,52% | 21,36% |
| Poland | 66,89% | 82,73% | 73,82% | 73,05% |
| Poland Caucasoid | 66,89% | 82,73% | 73,82% | 73,05% |
| Portugal | 66,14% | 76,70% | 65,92% | 64,19% |
| Portugal Caucasoid | 66,14% | 76,70% | 65,92% | 64,19% |
| Russia | 49,62% | 71,37% | 67,81% | 54,13% |
| Russia Caucasoid | 69,15% | 86,96% | 80,54% | 76,52% |
| Russia Other | 54,35% | 79,56% | 77,34% | 75,00% |
| Russia Siberian | 47,20% | 71,03% | 68,24% | 52,10% |
| Rwanda | 59,30% | 60,18% | 52,94% | 51,98% |
| Rwanda Black | 59,30% | 60,18% | 52,94% | 51,98% |
| Samoa | 64,65% | 71,25% | 69,80% | 41,48% |
| Samoa Polynesian | 64,65% | 71,25% | 69,80% | 41,48% |
| Sao Tome and Principe | 57,05% | 62,90% | 43,90% | 40,03% |
| Sao Tome and Principe Black | 57,05% | 62,90% | 43,90% | 40,03% |
| Saudi Arabia | 70,22% | 80,14% | 71,88% | 68,99% |
| Saudi Arabia Arab | 70,22% | 80,14% | 71,88% | 68,99% |
| Scotland | 63,88% | 89,95% | 82,44% | 80,82% |
| Scotland Caucasoid | 63,88% | 89,95% | 82,44% | 80,82% |
| Senegal | 28,43% | 30,28% | 26,56% | 18,82% |
| Senegal Black | 28,43% | 30,28% | 26,56% | 18,82% |
| Singapore | 44,94% | 62,42% | 56,57% | 49,02% |
| Singapore Austronesian | 44,94% | 62,42% | 56,57% | 49,02% |
| Slovenia | 70,84% | 83,68% | 72,71% | 71,98% |
| Slovenia Caucasoid | 70,84% | 83,68% | 72,71% | 71,98% |
| South Africa | 32,10% | 32,10% | 7,65% | 5,91% |
| South Africa Black | 32,10% | 32,10% | 7,65% | 5,91% |
| South America | 36,58% | 57,49% | 51,45% | 28,94% |
| South Asia | 62,00% | 73,68% | 64,18% | 62,40% |
| Southeast Asia | 45,96% | 54,23% | 48,55% | 35,91% |
| Southwest Asia | 36,93% | 43,05% | 35,64% | 35,00% |
| Spain | 67,50% | 79,74% | 69,61% | 68,76% |
| Spain Caucasoid | 67,55% | 80,07% | 70,00% | 69,16% |
| Sudan | 54,30% | 59,30% | 39,78% | 39,78% |
| Sudan Mixed | 54,30% | 59,30% | 39,78% | 39,78% |
| Sweden | 67,85% | 86,67% | 80,66% | 65,95% |
| Sweden Caucasoid | 67,85% | 86,67% | 80,66% | 65,95% |
| Thailand | 53,35% | 62,97% | 55,89% | 40,63% |
| Thailand Oriental | 53,35% | 62,97% | 55,89% | 40,63% |
| Tokelau | 20,79% | 20,79% | 20,79% | 13,51% |
| Tokelau Polynesian | 20,79% | 20,79% | 20,79% | 13,51% |
| Tonga | 59,04% | 62,79% | 62,79% | 40,71% |
| Tonga Polynesian | 59,04% | 62,79% | 62,79% | 40,71% |
| Tunisia | 63,78% | 72,89% | 60,17% | 58,41% |
| Tunisia Arab | 64,31% | 73,11% | 59,44% | 58,31% |
| Tunisia Berber | 63,40% | 74,47% | 66,09% | 62,15% |
| Turkey | 64,00% | 74,40% | 64,24% | 63,03% |
| Turkey Caucasoid | 64,00% | 74,40% | 64,24% | 63,03% |
| Ukraine | 33,58% | 50,64% | 50,64% | 50,64% |
| Ukraine Caucasoid | 33,58% | 50,64% | 50,64% | 50,64% |
| United States | 66,71% | 86,68% | 76,89% | 73,26% |
| United States Amerindian | 21,28% | 42,43% | 40,68% | 22,31% |
| United States Asian | 65,04% | 76,13% | 70,55% | 56,76% |
| United States Austronesian | 50,97% | 55,60% | 51,67% | 40,57% |
| United States Black | 61,36% | 67,47% | 59,07% | 55,09% |
| United States Caucasoid | 69,05% | 89,12% | 79,43% | 78,04% |
| United States Hispanic | 52,03% | 71,86% | 64,18% | 52,00% |
| United States Mestizo | 49,80% | 71,47% | 64,00% | 49,71% |
| United States Polynesian | 56,98% | 66,91% | 56,98% | 49,13% |
| Vietnam | 45,51% | 52,57% | 44,04% | 27,24% |
| Vietnam Oriental | 45,51% | 52,57% | 44,04% | 27,24% |
| West Africa | 57,52% | 63,76% | 57,74% | 49,48% |
| West Indies | 57,91% | 65,52% | 60,95% | 57,16% |
| Zimbabwe | 61,93% | 62,18% | 53,08% | 51,00% |
| Zimbabwe Black | 61,93% | 62,18% | 53,08% | 51,00% |
