# Supplementary figures and images for: Population-Predicted MHC Class II Epitope Presentation of SARS-CoV-2 Structural Proteins Correlates to the Case Fatality Rates of COVID-19 in Different Countries
Source: Int J Mol Sci. 2021 Mar 5;22(5):2630. doi: 10.3390/ijms22052630 (PMC7961590; doi:10.3390/ijms22052630)

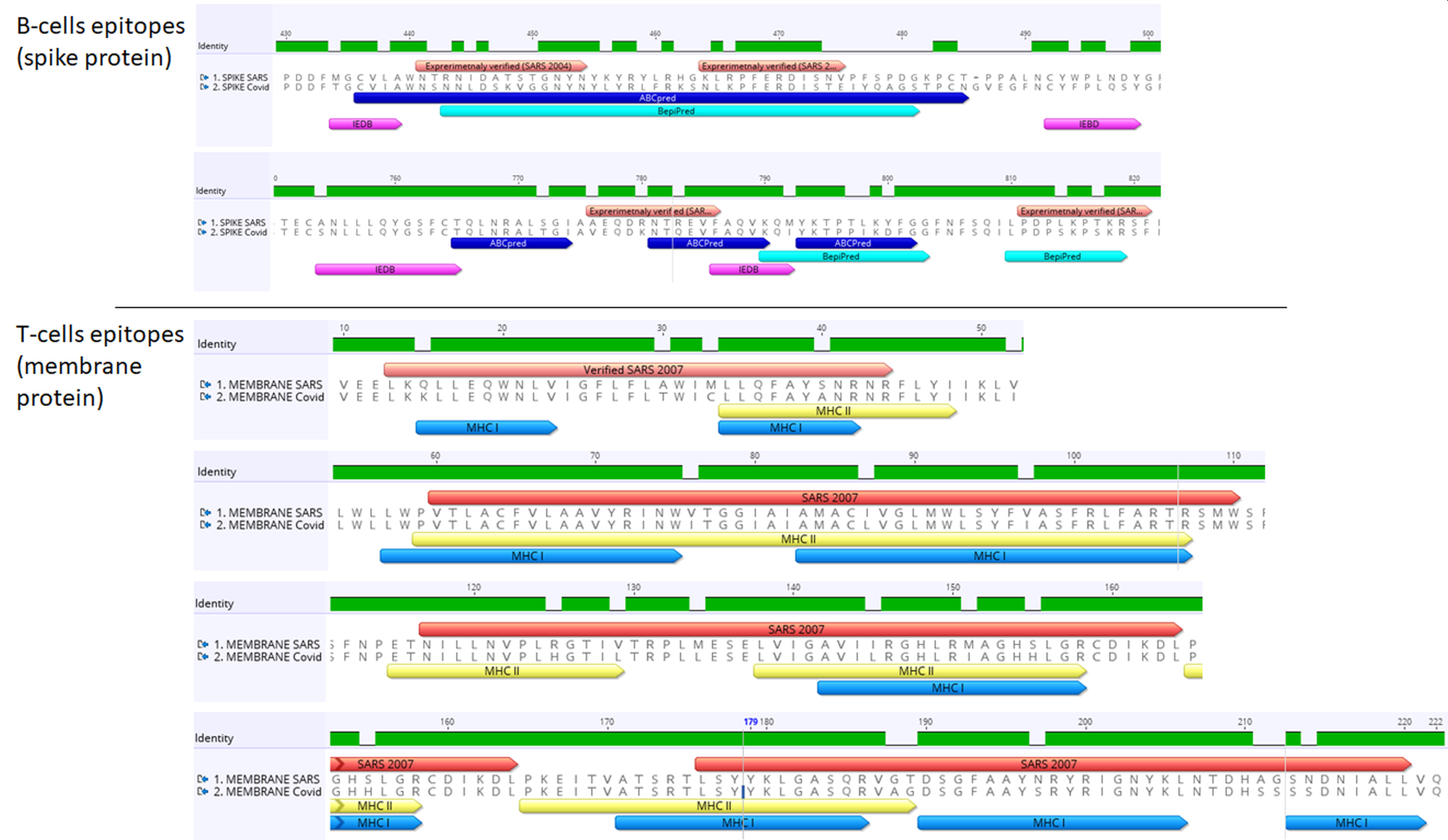

Supplement: Supplementary file 1 [file ijms-22-02630-s001.zip › FigureS1.png]
